# Exploring the larval fish community of the central Red Sea with an integrated morphological and molecular approach

**DOI:** 10.1371/journal.pone.0182503

**Published:** 2017-08-03

**Authors:** Stamatina Isari, John K. Pearman, Laura Casas, Craig T. Michell, Joao Curdia, Michael L. Berumen, Xabier Irigoien

**Affiliations:** 1 Red Sea Research Center, Biological and Environmental Sciences and Engineering Division, King Abdullah University of Science and Technology, Thuwal, Kingdom of Saudi Arabia; 2 AZTI—Marine Research, Herrera Kaia, Portualdea z/g, Pasaia (Gipuzkoa), Spain; 3 IKERBASQUE, Basque Foundation for Science, Bilbao, Spain; Department of Agriculture and Water Resources, AUSTRALIA

## Abstract

An important aspect of population dynamics for coral reef fishes is the input of new individuals from the pelagic larval pool. However, the high biodiversity and the difficulty of identifying larvae of closely related species represent obstacles to more fully understanding these populations. In this study, we combined morphology and genetic barcoding (Cytochrome Oxidase I gene) to characterize the seasonal patterns of the larval fish community at two sites in close proximity to coral reefs in the central-north Red Sea: one shallower inshore location (50 m depth) and a nearby site located in deeper and more offshore waters (~ 500 m depth). Fish larvae were collected using oblique tows of a 60 cm-bongo net (500 μm mesh size) every month for one year (2013). During the warmer period of the year (June-November), the larval fish stock was comparable between sampling sites. However, during the colder months, abundances were higher in the inshore than in the offshore waters. Taxonomic composition and temporal variation of community structure differed notably between sites, potentially reflecting habitat differences, reproductive patterns of adults, and/or advective processes in the area. Eleven out of a total of 62 recorded families comprised 69–94% of the fish larval community, depending on sampling site and month. Richness of taxa was notably higher in the inshore station compared to the offshore, particularly during the colder period of the year and especially for the gobiids and apogonids. Two mesopelagic taxa (*Vinciguerria* sp. and *Benthosema* spp.) comprised an important component of the larval community at the deeper site with only a small and sporadic occurrence in the shallower inshore waters. Our data provide an important baseline reference for the larval fish communities of the central Red Sea, representing the first such study from Saudi Arabian waters.

## Introduction

Coral reefs are key ecosystems in tropical seas, providing food and resources to coastal populations [1] and epitomizing biodiversity with thousands of species sharing a limited space [2]. Coral reefs are fragile ecosystems, dependent not only on the framework provided by the corals, but are also reliant on a delicate equilibrium of species composition. Fishing pressure, in addition, can have unpredictable effects on the overall ecosystem function, with flow-on effects of changes in the community structure (i.e., trophic cascades), particularly when certain guilds such as herbivores or top predators are eliminated from the system [3, 4]. Specifically, overfishing poses a major threat in many coral reef systems [5], and management efforts are increasingly utilizing marine protected areas (MPAs) as a mitigation tool. In many cases, MPA design relies on modeled assumptions about fish early life-history stages and related factors (e.g., connectivity), especially where local empirical data are lacking [6, 7]. Therefore, a better knowledge of parameters related to the dynamics of fish populations, such as spatiotemporal larval distribution patterns, larval pool composition and seasonal recruitment, could help to improve our understanding of the factors affecting the community composition and potential resilience to fishing pressure.

Dramatic morphological changes during ontogeny make larval fish identification to the species level a substantial challenge. This is further complicated by the high diversity encountered in tropical waters and in the vicinity of reef habitats [8]. Even for larval fish experts, morphological identification of tropical fish taxa can be confusing and ambiguous even at a family level, particularly at the earlier developmental stages [9]. The combination of high species richness and a paucity of diagnostic morphological characteristics for larval life stages of reef fishes has been a notable obstacle in research efforts so far [8]. Although numerous assemblage studies of early-stage fish larvae (i.e., collected by towing planktonic nets) exist for tropical coral reef-associated waters in both the Indo-Pacific and western Atlantic [10–20], these have been mostly restricted to the family level and information at a lower taxonomic level (genus/species) has been rarely provided.

The Red Sea is a coral reef-dominated environment with a highly diverse ichthyofauna [21] and prominent endemism [22]. Although in recent years there has been increasing interest in the Red Sea and regional reef ecology [23, 24], the early life-history stages of fishes remain highly understudied in the region. Only a few works have dealt with juvenile stages, exploring the duration of the pelagic larval phase through otolith micro-chemistry and assessing the recruitment patterns for certain reef species [25–27]. Studies at the early life-history stages employing multispecies approaches have been almost absent. Presumably, limited reference material to identify local ichthyoplankton species has been one of the major bottlenecks. Molecular approaches (i.e., DNA barcoding), however, are a promising tool in estimating marine biodiversity, offering the opportunity for rapid and accurate species identification at all developmental stages and additionally revealing cryptic and sibling species [28]. The efficacy of this technique for the identification of the early life-history stages (eggs and larvae) has been verified in a variety of fish species and families from different environments [29–31], including coral reefs [20, 32, 33].

Here, we present the first data on the ecology of early planktonic developmental stages of fish in the central Arabian Red Sea. Utilizing a combination of morphological diagnostic criteria and DNA barcoding we determined the species of larval fish and their assemblage variation throughout an annual cycle in two sampling stations near coral reefs. Our main objective was to determine the agents (e.g., abiotic, biotic) that have potentially shaped the spatiotemporal abundance patterns of fish larvae in the study area, providing in parallel baseline knowledge of the diversity and assemblage structure of larval fish and informing future large-scale studies of recruitment dynamics.

## Materials and methods

### Ethics statement

The research was undertaken in accordance with the policies and procedures of the King Abdullah University of Science and Technology (KAUST). Permits for sampling in Saudi Arabian waters were obtained from the Saudi Arabian coastguard. No specific permissions were required, as the study did not involve endangered or protected species.

### Ichthyoplankton and hydrographical sampling

**Fig 1:**
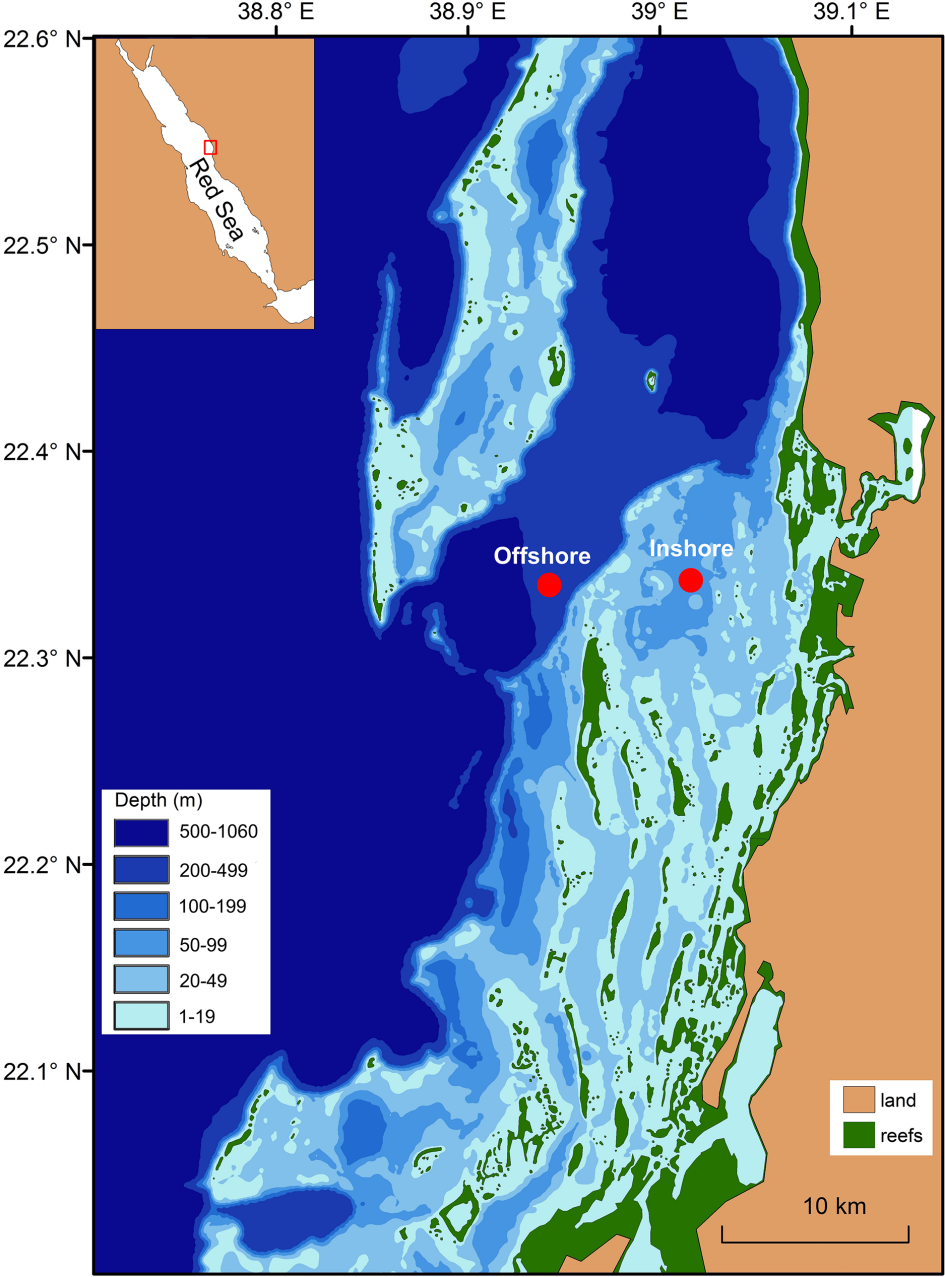
Map of the study area. Geographical location of the study area and distribution of sampling sites. The inshore station (22°19'25.22"N, 39°0'47.97"E) was located ca. 10 km from the coast and 8 km from the offshore station (22°18'18.48"N, 38°56'4.51"E). Credit for bathymetric map: Maha Khalil.

Ichthyoplankton was collected monthly in 2013 (from January to December) near Thuwal, a coastal area in the central Saudi Arabian Red Sea with an extensive network of coral reefs. Two distinct sites in close proximity to coral reefs were sampled once per month: one shallow inshore station (ca. 50 m depth) and a nearby offshore, deeper station (~500 m depth) (Fig 1). Overall, we sampled between the first quarter and the full moon (ca. the 3^rd^ week of each month, S1 Table), primarily due to logistical constraints. Sampling was always undertaken during daylight hours (between 09:00–11:00 hrs with one tow per each sampling site) following recommended procedures for ichthyoplankton collection [34], using oblique tows of a 60 cm-bongo net (500 μm mesh size) at 2–2.5 knots from 50 m depth to the surface. Haul depth was monitored onboard constantly during the tow using a CTD mounted 1 m above the net. The volume of filtered seawater (on average 210 m^3^ per tow) was calculated from a calibrated digital flowmeter (model 23.090, KC Denmark) mounted at the mouth of each net. Samples were immediately preserved in 98% ethanol (Sigma-Aldrich, St. Louis, MO) after collection and stored at 4 ^o^C. To describe the thermohaline structure of the water column, vertical CTD deployments were performed with an Idronaut Ocean seven 316Plus profiler.

### Fish larval sorting and morphological identification

All fish larvae were sorted from the plankton samples. Due to the small number of larvae distributed across the distinct developmental stages (e.g., preflexion, flexion, postflexion), all larvae per taxonomic category were pooled together and their abundance was standardized as a number per square meter. Pooling all the developmental stages would additionally minimize the influence of the lunar phase in the temporal patterns of fish larval abundance. Zooplankton displacement volume (ZDV) was also measured for each sampling site according to Smith and Richardson [34] and used as a rough estimation of zooplankton biomass. The vast majority of the larvae were identified at least to the family level by morphological approaches [35–39], except for a minor fraction (0.7% of the total) involving yolksac stages and damaged larvae. Larval fish were further distinguished into two main categories: larvae of the family Gobiidae (a major component of community, see Results section) and non-gobiid larvae including the rest of the families. Identification below the family level was carried out using a combination of morphological characters and DNA barcoding techniques.

### Molecular larval fish identification

Mitochondrial Cytochrome c Oxidase subunit I (COI)-barcoding served as a tool to extend the taxonomic identification to the sub-family level. The COI gene, due to its low intraspecific but high interspecific genetic variation, has been considered an efficient species-specific marine metazoan molecular marker [28] and is typically effective for discrimination of fish species [40].

A total of 2115 larvae (46% of the total collected), including 1149 non-gobiids (43% of total non-gobiids) and 966 gobiids (52% of the total gobiids), were selected for molecular analysis. The number of processed larvae per sample depended on the morphological diversity encountered and ranged from 21 to 85% for the non-gobiids and from 33 to 83% of gobiids (S1 Fig). Larvae with the same morphological characteristics were repeatedly processed across different samples to verify the accuracy of the morphological identification. In the case of the family Gobiidae, although several morphotypes could be distinguished based on body shape and pigmentation pattern, a good match between morphology and barcodes was not always possible (some gobiid morphotypes presented a high molecular diversity). Hence, a subsample of larvae in each morphotype per sample was arbitrarily chosen for barcoding. The relative molecular species composition encountered was then extrapolated to the rest of the non-processed larvae of the same morphotype in the sample.

#### DNA extraction, PCR amplification and sequencing

Whole genomic DNA was extracted from larval fish individuals (after being photographed) using the DNeasy Blood and Tissue extraction kit (QIAGEN). COI amplification was done using the M13-tailed primers FishF1t1–C_FishR1t1 (Fish cocktail, S2 Table) with suggested concentrations for PCR reagents and template DNA [41]. The thermocycler profile was 94°C for 2 min, six cycles of 94°C for 30 s, 49°C for 40 s and 68°C for 30 s, followed by 35 cycles of 94°C for 30 s, 52°C for 40 s and 68° C for 1 min, with a final extension at 68°C for 10 min. PCR products were purified with Illustra^TM^ ExoStar^TM^-1 Step at 37°C for 20 min, followed by 80°C for 15 min. Sanger sequencing was performed on an ABI 3730 XL DNA analyzer at the Bioscience Core Lab facilities (KAUST). SeqTrace software [42] was used to pair forward and reverse sequence reads and check their quality (i.e., removing unreliable bases).

#### Species assignment

Operational taxonomic units (OTUs) were constructed from the processed reads at a 97% similarity level and were further compared against the non-redundant nucleotide (nt) database of the National Center for Biotechnology Information (NCBI) using the Blast algorithm [43] within the software Geneious 9.0.5 [44]. We additionally blasted our results against an internally curated Red Sea reference library of COI sequences, built based on barcode deposits from juvenile and adult fish collected from Arabian Red Sea reefs (submitted to GenBank with Accession Numbers KU191051 to KU191598). Sequences which had a sequence similarity greater than 97% were assigned to the species level using the top Blast hit. For those specimens where the 97% threshold was not met, attempts to assign sequences to an appropriate taxon were additionally made using the Statistical Assignment Package (version 1.9.6.1) [45] against the NCBI database using a minimum identity of 0.8 and a default posterior probability of 95%. Species names were confirmed in the World Register of Marine Species (WoRMS) [46] and the presence of species in the Red Sea was checked in the list of Red Sea Fishes [21] and FishBase [47]. For the purposes of this work and coherence with WoRMS, all “Scarinae labrid” species have been considered as Scaridae. A phylogenetic tree for the Apogonids was created in Mega (V6) using the maximum likelihood algorithm. The Jukes Cantor model was used with partial deletion and the tree was bootstrapped (n = 1000). All sequences have been deposited in GenBank (BankIt1984376 with accession numbers KY675398—KY676194).

### Community structure analysis

Multivariate techniques in PRIMER v7 software [48] were used to compare larval fish community structure among samples. Abundances of the identified taxa (standardized to m^2^) were square root transformed and a similarity matrix based on the Bray-Curtis similarity index was then constructed and subjected to hierarchical agglomerative clustering (group average linkage) and non-metric multidimensional scaling (NMDS) ordination [49]. Associations among larval taxa with a relative density >3% in at least one sample were further explored by calculating an “index of association” (i.e., Bray-Curtis similarity index on pairs of taxa based on standardized abundances), followed by agglomerative hierarchical clustering [49]. The statistical significance of sample clustering was tested with the similarity profile routine (SIMPROF) at 1% significance level [50]. Community diversity in space and time was assessed using the following calculations for each sample: species richness expressed as total number of species present (S), Shannon-Wiener species diversity index (H΄) and Pielou’s species evenness index (J΄).

To relate community structure with environmental parameters, ordination scores produced by the NMDS were compared by multiple regression analysis with measured parameters in our study (i.e., zooplankton displacement volume, mean temperature and salinity in the sampling layer) to determine which of them might best explain the fish larvae distributions [51, 52]. In the regression analysis, the NMDS scores were treated as the independent variables and each environmental parameter as the dependent variable. More details and discussion of the advantages of the method are provided in Somarakis et al. [53]. Regression lines and their directions were plotted in the NMDS graphs according to Kruskal and Wish [51]: the direction of maximum correlation of each regression line is at an angle *φ*_r_ with the r^th^ MDS axis. The direction cosine, or regression weight c_r_, of that angle is given by the formula:

$$c_{r}=b_{r}/\sqrt{b_{1}^{2}+b_{2}^{2}}$$

where b_1_ and b_2_ are the coefficients from the multiple regression a+b_1_x_1_+b_2_x_2_ and x_1_ and x_2_ are the scores in the first and second MDS axis respectively.

## Results

### Environmental conditions

**Fig 2:**
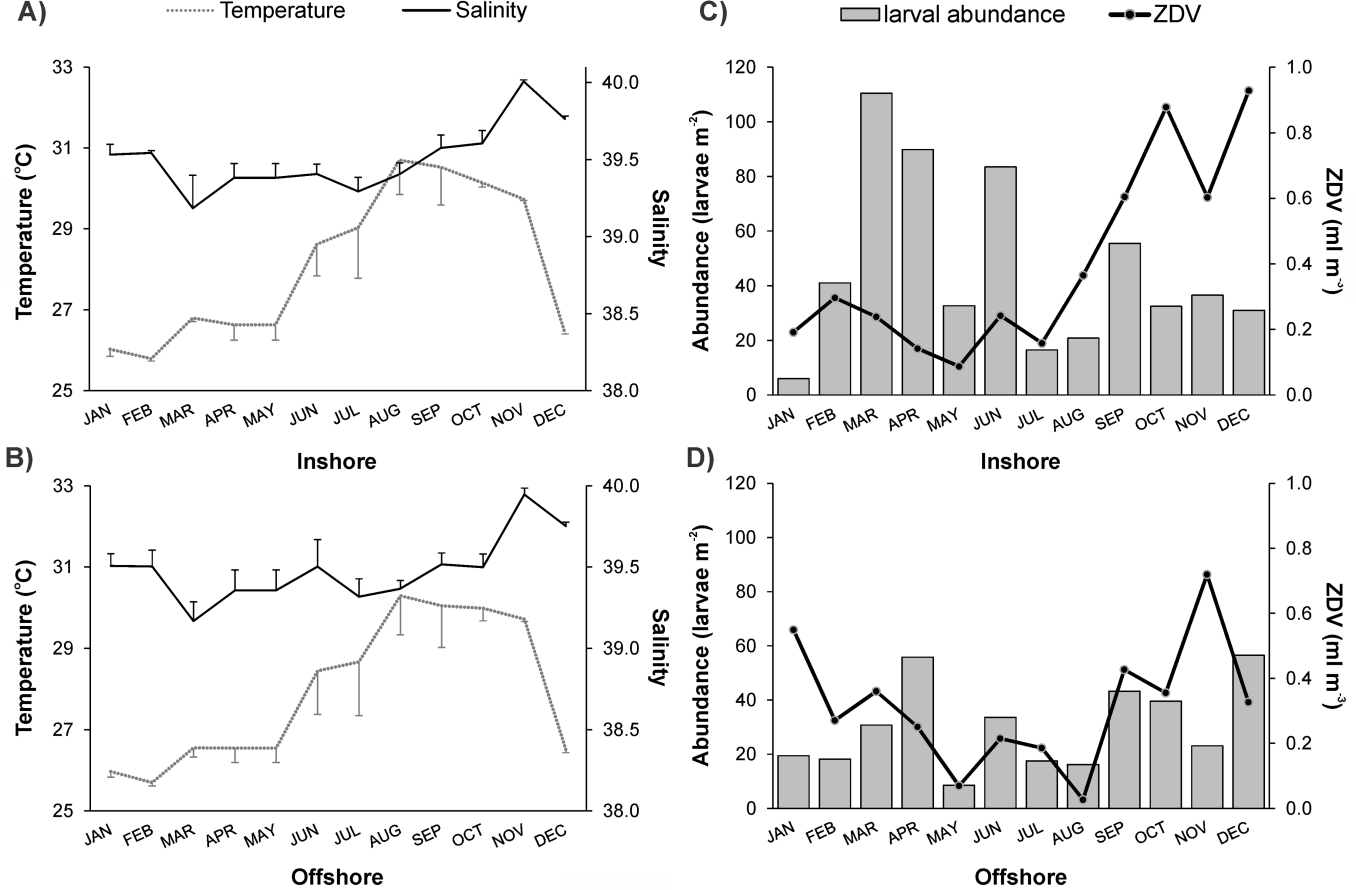
Monthly variation of the environmental parameters in the sampling layer. A) Average of temperature (^o^C) and B) salinity in the 0–50 m layer for every month and site (error bars indicate the standard deviation). C) Monthly variation of zooplankton displacement volume (ZDV, ml m^-3^) and D) larval abundance (larvae m^-2^) in the inshore and offshore site.

Environmental conditions (temperature, salinity and ZDV) in the sampling layer (0–50 m) showed similar variation in both sites throughout the year cycle (Fig 2). Average temperature in the upper 50 m of the water column revealed a strong seasonal pattern (Fig 2A and 2B). Lower temperatures were recorded in the winter-spring months with minima in February (25.7 ^o^C) and higher values during summer-autumn with maxima in August (max 30.7^°^C). Thermal stratification of the water column became prominent by the end of spring (as reflected by higher standard deviation values) with the thermocline initiating at 15–20 m depth; mixing processes in the upper layer were evident after October (Fig 2A and 2B). Salinities ranged from 39.2 to 40.01, with higher values during the winter (Fig 2A and 2B). A prominent increase of ZDV values was observed at both stations after August, though this was stronger at the inshore site (Fig 2C and 2D). ZDV variability during the colder months of the year was higher offshore.

### Fish larval abundance and family composition

A total of 4566 were sorted from the collections (2577 larvae from the shallow site, 1811 from the deeper site). Annual average larval abundance did not differ significantly between sampling sites (t-test, t = -1.565, P = 0.132), but the monthly variation differed considerably (Fig 2C and 2D). Total abundance at the inshore station increased remarkably during the colder period of the year (winter-spring) reaching a maximum of 110 larvae m^-2^, while notably lower values were recorded in January (6 larvae m^-2^). No clear seasonal pattern was observed in the offshore waters, where the spring abundance maximum (56 larvae m^-2^) was comparable to the other months.

**Fig 3:**
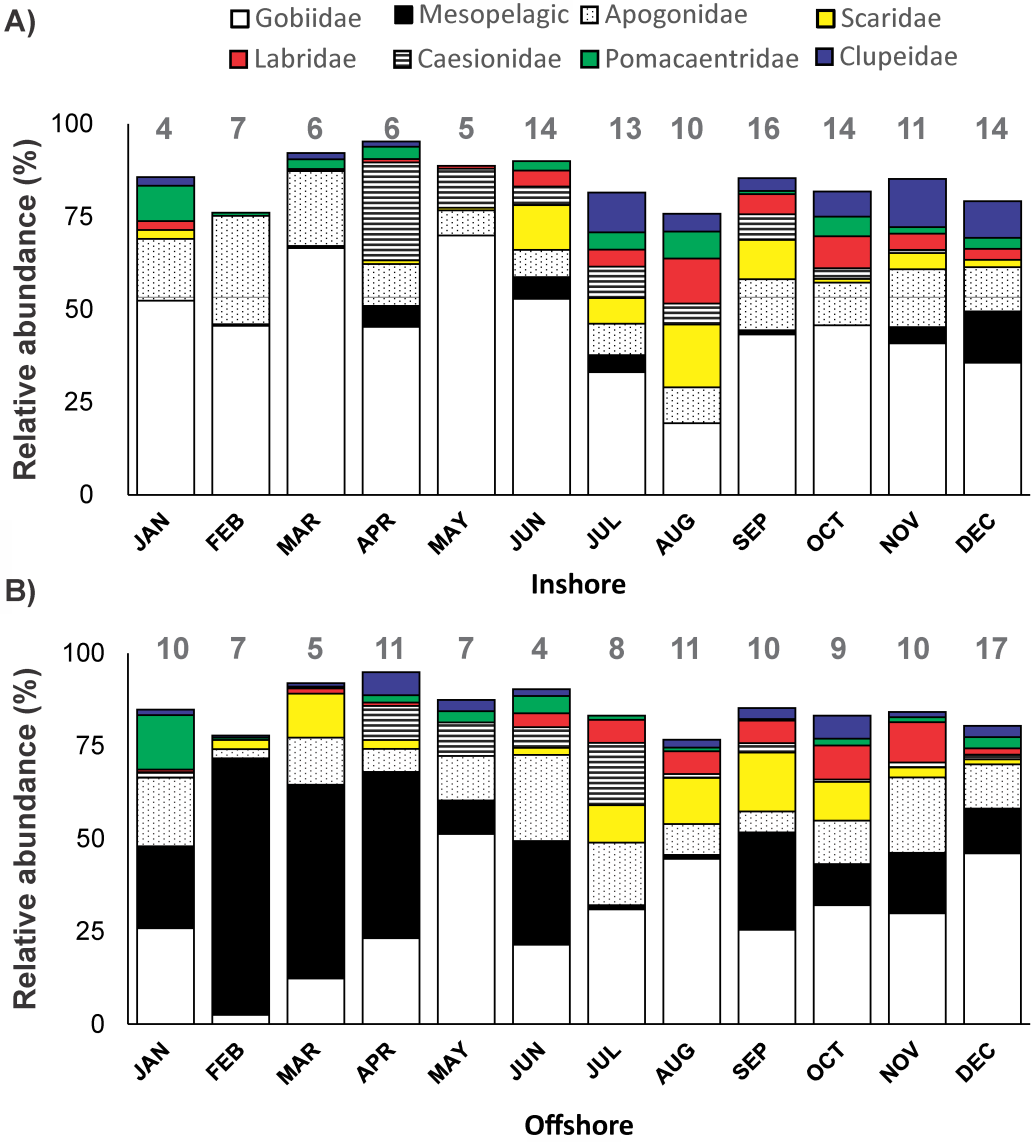
Relative abundance of the major families. The % contribution of the eleven major families in the monthly collections of the inshore (A) and offshore (B) station. Mesopelagic category includes species belonging to the families Phosichthyidae, Myctophidae, Bregmacerotidae, Paralepididae and Stomiidae. Values on the top of each bar correspond to the numbers of the rest of the families encountered in the samples.

**Fig 4:**
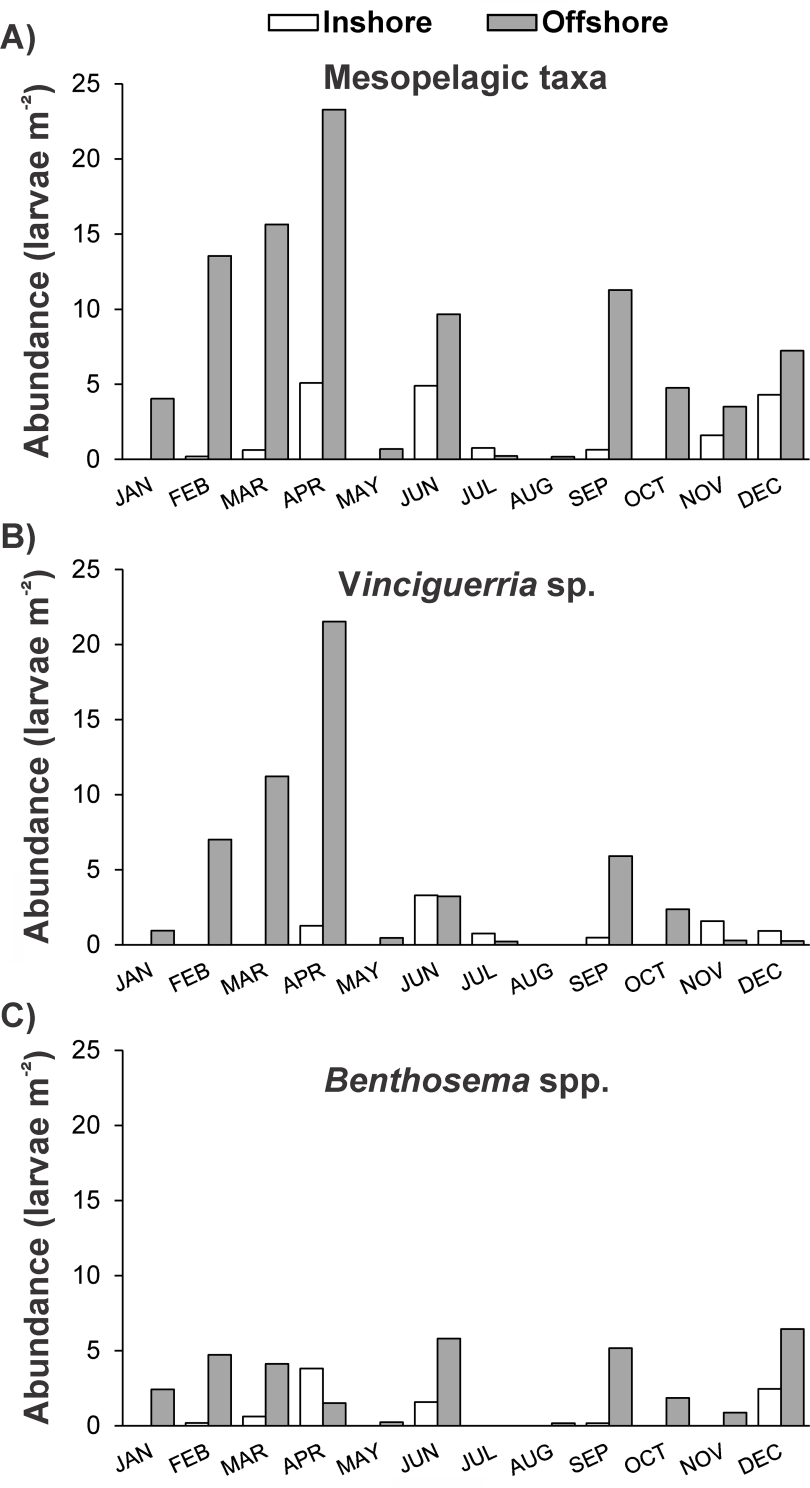
Abundance of mesopelagic fish larvae. (A) Monthly abundance values (larvae m^-2^) of all mesopelagic families (Phosichthyidae, Myctophidae, Bregmacerotidae, Stomiidae, Paralepididae) encountered in the samples of both stations. Abundances of the taxa *Vinciguerria* sp. (B) and *Benthosema* spp. (C) are also provided.

**Fig 5:**
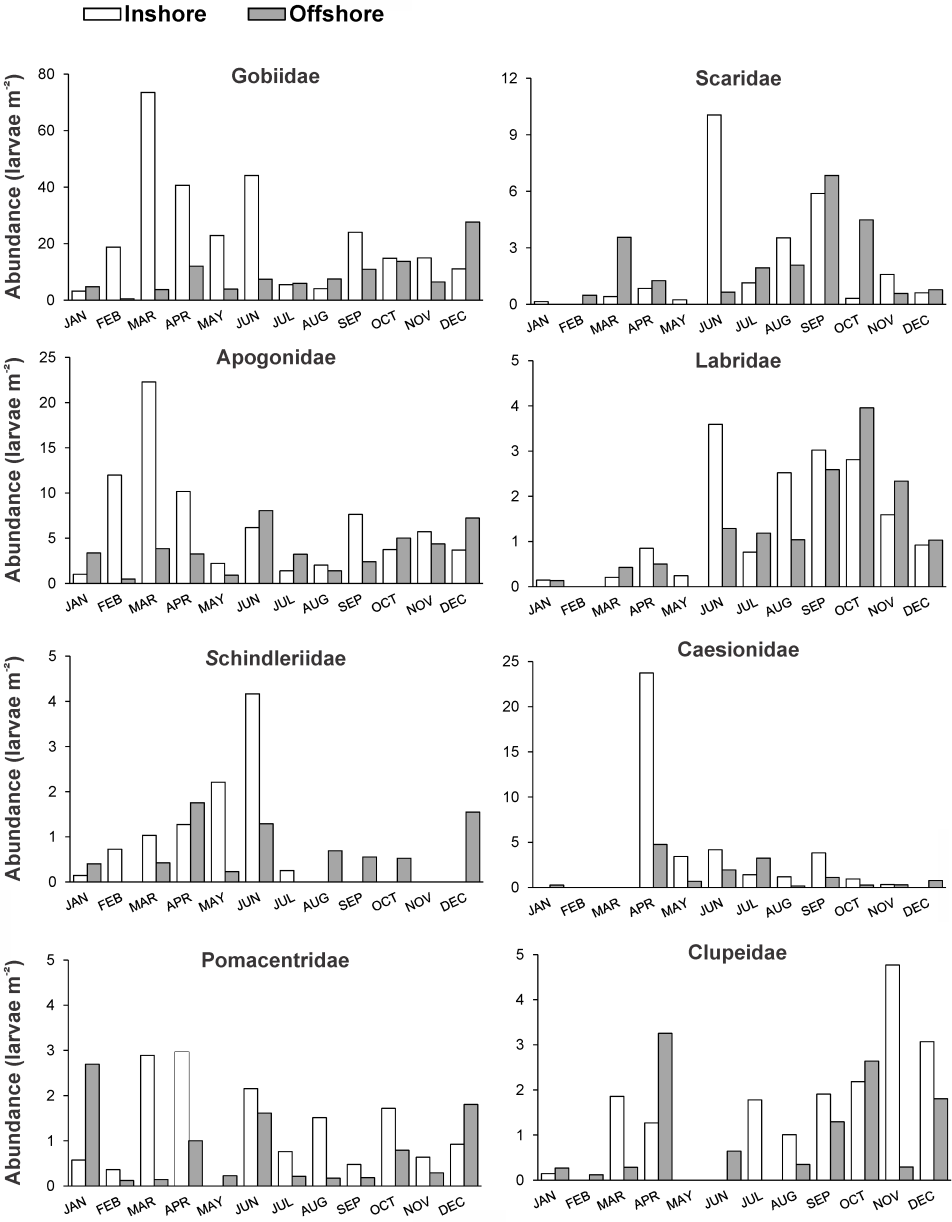
Abundance of major families. Monthly abundance values (larvae m^-2^) of major families in the two sampling sites (inshore, offshore).

A total of 62 families belonging to 16 orders were identified in the collections (S3 and S4 Tables, S2 Fig). Eleven families dominated the larval fish community accounting for 69–94% (Fig 3), while half of the remaining families occurred only sporadically with negligible contributions (<1%, S2 Fig). Family composition presented considerable differences in space and time. Larvae of mesopelagic families (mainly Phosichthyidae and Myctophidae), dominated the offshore community particularly during the colder months of the year (up to 75% of the sampled individuals), with only minor abundances in inshore waters (Figs 3 and 4). Gobiid and apogonid larvae were the main components of the community inshore (70% and 29%, respectively) and offshore (49% and 24%, respectively), but with notably higher inshore presence in early spring (peak in March) (Fig 5). Interestingly, contrasting abundance patterns were observed for the families Scaridae and Labridae; in both stations higher larval abundances were recorded in the warmer period (peak September-October) accounting for up to 17% and 12% of the community, respectively. Other families (e.g., Caesionidae, Pomacentridae and Clupeidae) with an important relative contribution in the larval fish community, in most cases presented higher abundances after spring time (Fig 5).

### Species assignment: DNA barcoding and morphology

Based on both genetics and morphology a total of 321 species were identified in our larval samples. At a sub-family level, we identified a total of 308 OTUs, 209 corresponding to non-gobiid families (S3 Table) and 99 to gobiids (S4 Table). Additionally, 13 non-gobiid taxa (belonging to 13 families S3 Table) where identified based only on morphological criteria (sequences were not successfully obtained). Blasting of the non-gobiid COI barcodes to the NCBI database resulted in assignments to species and genus level for 68% of the cases (species: 60%, genus: 8%); for the rest of the sequences it was not possible to assign a specific name, although we still considered them as distinct “genetic species” within their families. Among the non-gobiid taxa, the limitations of the reference databases were particularly evident for the Apogonidae, where 64% of the barcodes did not match a reference sequence. In the case of gobiid barcodes, only 21% of the OTUs matched sequences within the reference databases.

Sequences were not successfully obtained for the entire number of larvae processed molecularly (likely due to individual DNA degradation processes in some of the samples and primer mismatch for certain taxa) (S1 Fig). However, successful COI-barcoding in 32% of the total non-gobiids collected, with numerous repetitions of the same morphotypes, allowed taxonomic identification based on morphology (matched with photos of successfully barcoded specimens) for the majority of larvae encountered in the samples (96% of the non-gobiids). In contrast, barcoding was only successful in 41% of the gobiids, and even when combined with identification based on morphological criteria, only 55% of total gobiids could be identified, so an extrapolation had to be applied to estimate relative abundance for the rest of the larvae (S1 Fig).

### Diversity indices and species composition

**Fig 6:**
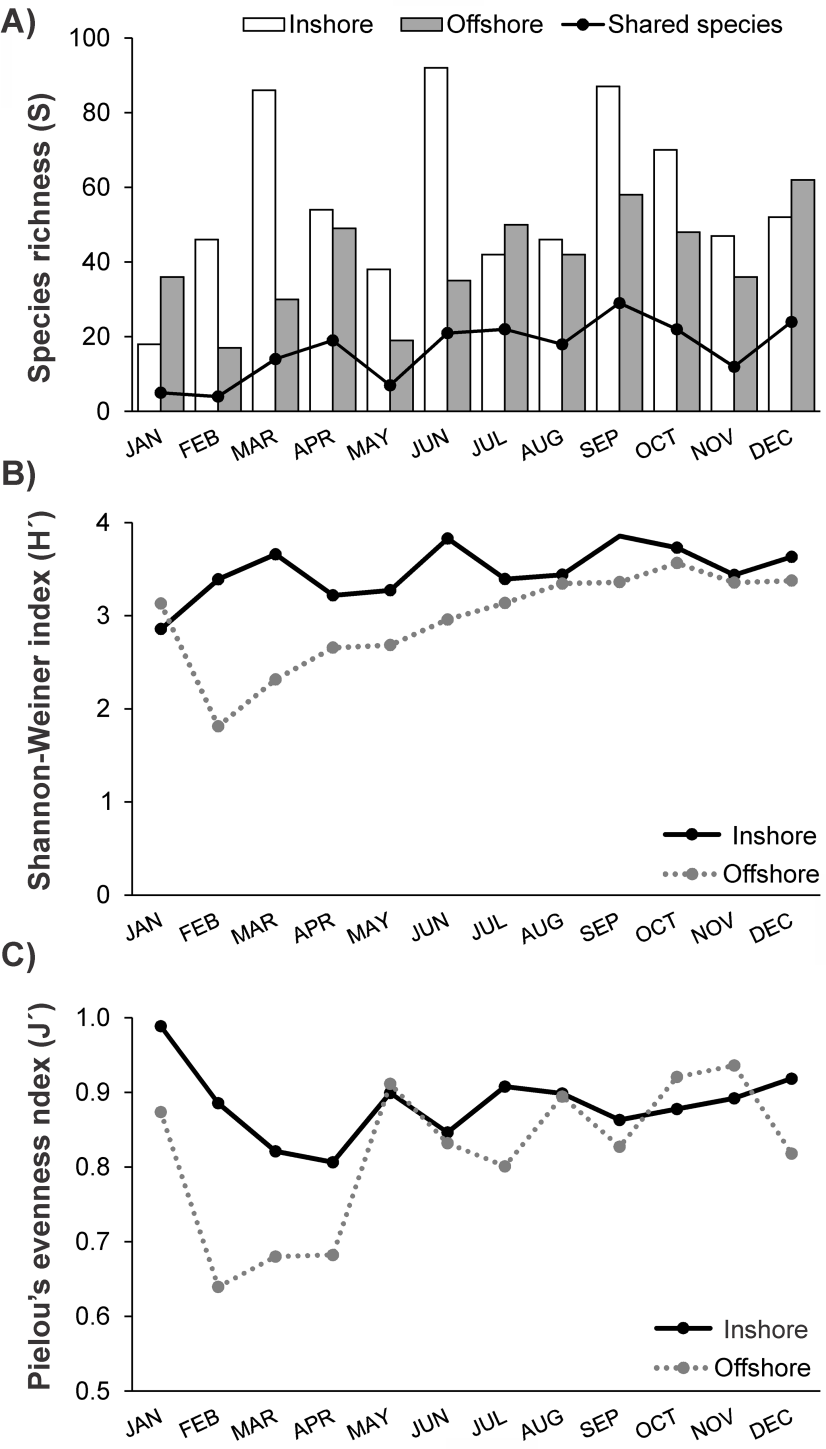
Diversity of larval fish. Species richness **(A)**, Shannon-Weinner (B) and Pielou’s (C) diversity indices of encountered taxa in the samples of both stations.

Diversity indices showed variability on a temporal and spatial scale (Fig 6). Overall, species richness was higher inshore (apart from Jan, Jul, Dec) compared to offshore, although a substantial number of species were found in both sites (203 species found offshore, 258 found in inshore samples, 140 shared between the sites). Shannon and Pielou’s indices were higher inshore, but an increase during the year from a minimum in February was also evident in the offshore larval community. Notably low diversity values in February in offshore waters were mainly due to the absence of gobiids from the samples and the unsuccessful DNA barcoding for the few gobiids sampled (S1 Fig).

Species richness within families was almost double in the inshore shallow waters both for the gobiids (total: 99, offshore: 54, inshore: 91, shared: 46) and the apogonids (total: 39, offshore: 23, inshore: 36, shared: 20) (S3 Table). Gobiid fauna, mostly represented by species belonging to the genera *Eviota*, *Amblygobius*, *Koumansetta*, *Oxyurichthys* and *Amblyeleotris*, was notably enriched in the inshore waters in particular sampling months (i.e., March, June and September) with some species exclusively present at these sampling occasions (S3 Fig). Similarly to gobiids, apogonids showed higher species richness during spring time and June. Although some species were an important component throughout the year with a peak in spring time (i.e., Apogonidae sp. 22, Apogonidae sp. 9 and Apogonidae sp. 21, as well as *Fowleria marmorata* at the deeper site), others had a restricted occurrence before or after the warm period of the year (S4 Fig).

The rest of the families had lower species richness levels and assignment to species/genus level was possible in most cases (S3 Table). The spatiotemporal patterns in species abundance for the major families are presented in S5 Fig. The family Labridae included 20 species (deep site: 15, shallow site: 15, shared: 10) with *Paracheilinus octotaenia* and Labridae sp. 1 being the most important in terms of abundance, while *Stegastes nigricans* was the main representative of the family Pomacentridae (total: 16, offshore: 9, inshore: 14, shared: 7). Scaridae included 8 species (deep site: 6, shallow site: 8, shared: 6); *Scarus niger* was present mostly in the colder period at the offshore site, while the warmer scarid assemblages mainly consisted of the species *Scarus cf*. *ferrugineus*., *Hipposcarus* sp. and *Chlorurus sordidus*. Clupeidae and Caesionidae were represented almost exclusively by the species *Spratelloides gracilis* and *Casio cf*. *caerulaurea*, respectively.

### Species assemblage structure and relation with the environment

**Fig 7:**
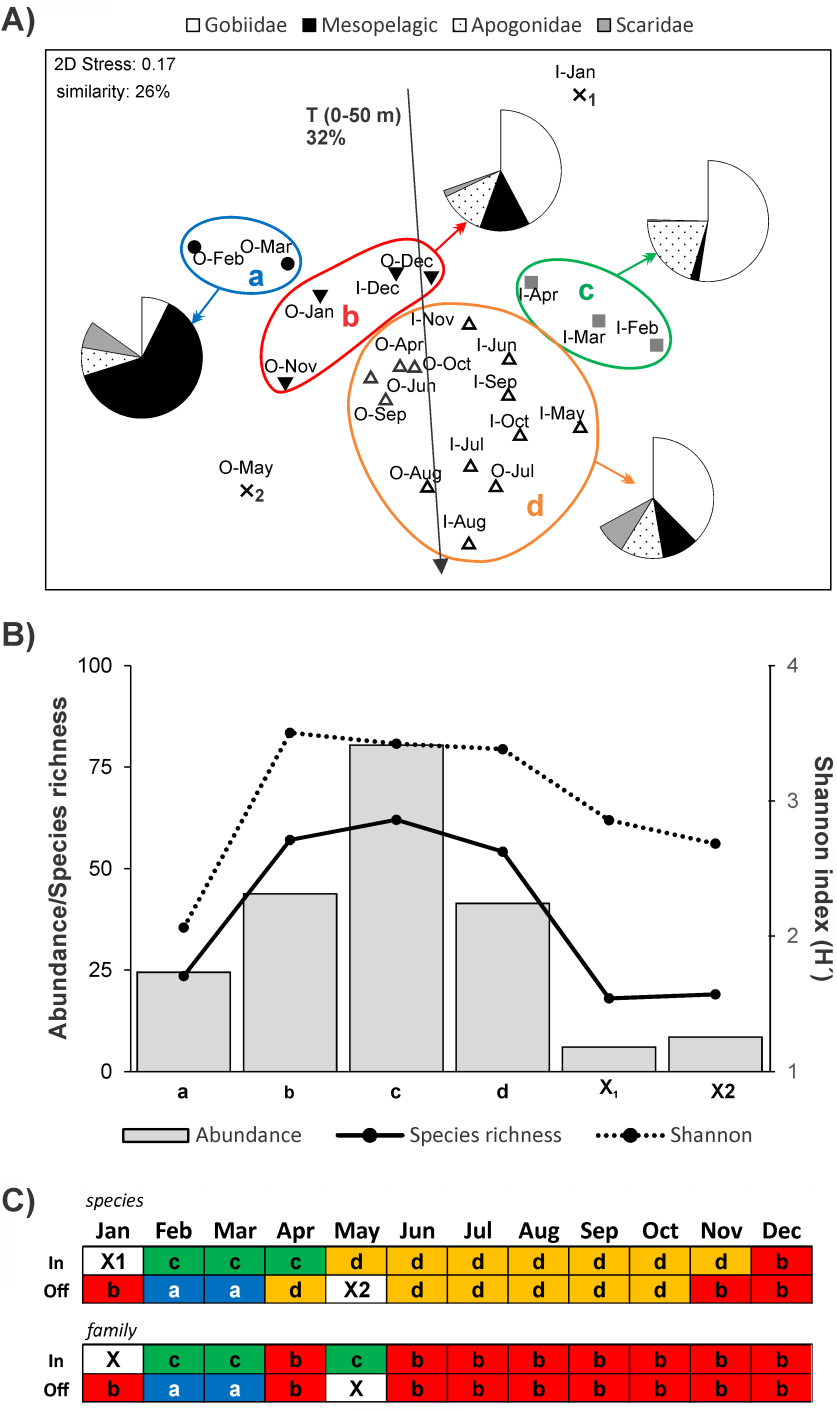
Grouping of samples based on larval fish community structure. (A) Ordination plot of the comparison between samples (I: inshore, O: offshore) using non-metric multidimensional scaling and Bray–Curtis similarity index. Cluster groups a-d identified at 26% similarity level are superimposed and mean relative contribution of major taxa (Gobiidae, Mesopelagic families, Apogonidae, Scaridae) in each group is presented in pies (missing part of the pie corresponds to remaining families). Significant multiple regression between ordination scores and average temperature at the sampling layer is shown, as well as the fraction (%) of variance in the fish larval data explained by this parameter. (B) Average larval abundance, species richness and Shannon’s index for the groups of samples identified by the cluster analysis (Groups a-d) (C) Schematic representation of cluster of samples based on species and family composition.

**Fig 8:**
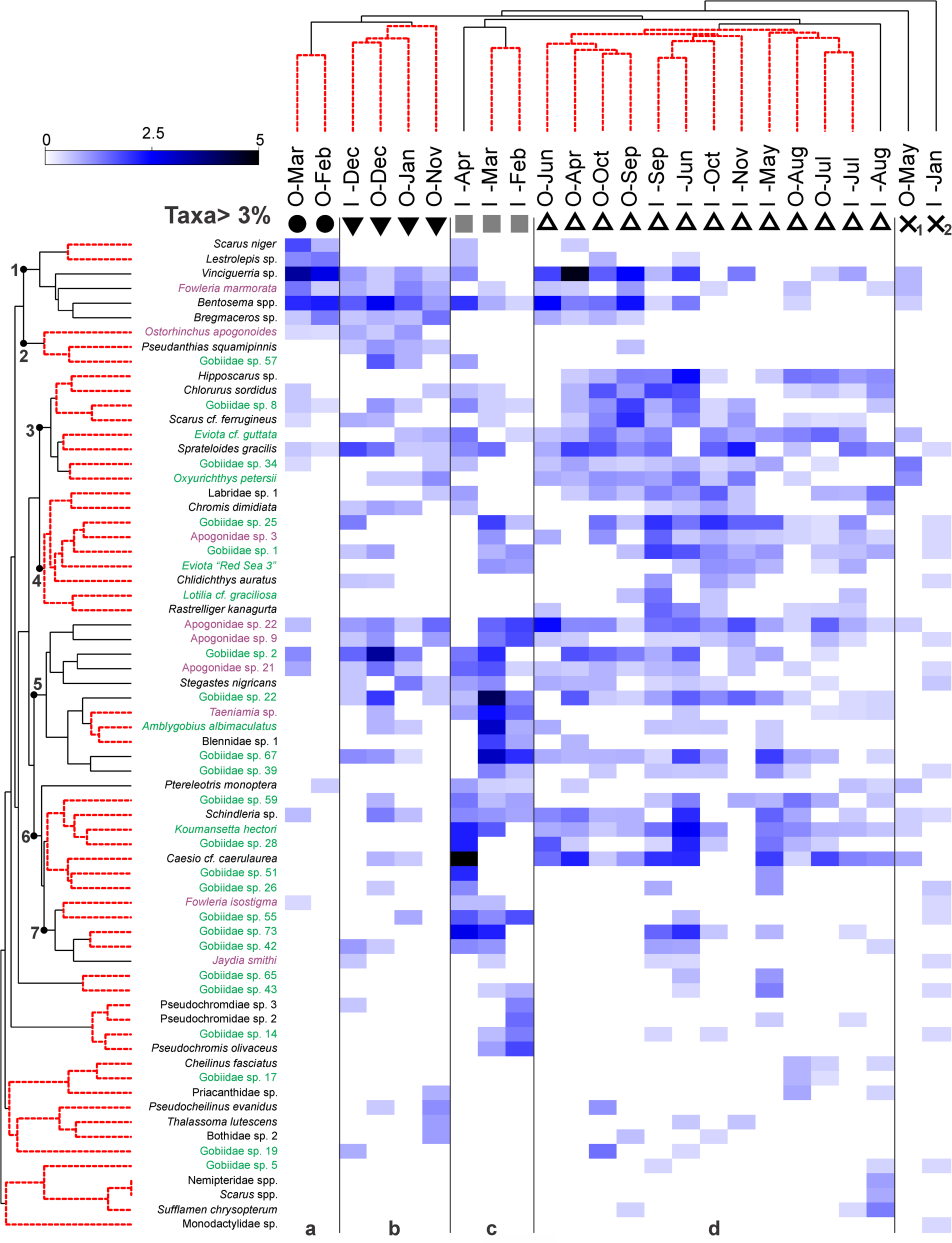
Major taxa contributing to sample grouping. Shade plot of square-root transformed abundances for taxa accounting >3% of the total abundance at least in one sample. Monthly collections have been grouped (Groups a-d) based on the total identified taxa. Linear color-scale is proportional to the square root transformed abundances of each taxon. Taxa of larval fish are also clustered (agglomerative, group average linkage) based on “index of association” resemblances computed on their standardized abundances. Species associations (assemblages) are indicated with numbers 1–7. Red-dashed lines in sample and species clusters indicate lack of further community structuring inside the specific clusters (according to SIMPROF at *p* 1%). Gobiid and apogonid taxa are presented in green and purple, respectively.

Multivariate analyses based on species composition revealed a considerable structuring in the larval community in space and time (Fig 7A). Cluster analysis at 26% similarity identified 4 main groups of samples (Groups a-d) that were largely verified by the SIMPROF analysis (Figs 7A and 8). Group a comprised offshore samples from early spring (i.e., Offshore-Feb, Offshore-Mar) with overall low abundance and diversity values (Fig 7A and 7B), as well as particular species assemblages (Fig 8: assemblages 1 and 2), including mesopelagic taxa (i.e., *Benthosema* spp., *Vinciguerria* sp., *Lestrolepis* sp. and *Bregmaceros* sp.), few demersal species (e.g., the scarid *Scarus niger* and the apogonid *Fowleria marmorata*) and the epipelagic *Spratelloides gracilis*. Group b was mainly comprised of samples collected offshore in the colder period of the year (Fig 7A). This group, besides including species associations similar to Group a, had the notable presence of several demersal species such as the serranid *Pseudathias squamipinnis* and various gobiid (e.g., *Priolepis cincta*, Gobiidae sp. 2) and apogonid species (Apogonidae sp. 9, Apogonidae sp. 22, Apogonidae sp. 18) (Fig 8). Collections from inshore waters during early spring (Inshore-Feb to Inshore-Apr) were separated (Group c) from the rest of the samples due to their increased and highly diversified larval stock (Fig 7A and 7B), mostly dominated by gobies, cardinalfishes and larvae of some other characteristic demersal species (e.g., *Schindleria* sp., Pseudochromidae spp.) (Fig 8: assemblages 5–7). Most of the remaining samples, collected during the warmer period of the year, were grouped together (Group d) (Fig 7A), likely due to increased presence of larval parrotfishes in the community. Finally, two samples with extremely low total abundances and diversity (i.e., Inshore-Jan and Offshore-May) were separated from all other groups (Fig 7A). For the sake of comparison, we repeated the same analysis but restricted our data to the family taxonomic level. The Group b could not be distinguished from Group d (Fig 7C).

Among the environmental parameters tested only the average temperature in the sampling layer could explain the variation in the NMDS ordination (Table 1). Based on relative angles of intersection, temperature seemed to be mostly related with the differentiation of Group d (samples collected during the warmer period) from all the others (Fig 7A).

**Table 1:** Association of environmental parameters with the larval fish community structure. Multiple regression analysis between environemntal parameters (T: average temperature in the 0–50 m layer, S: average salinity in 0–50 m layer, ZDV: zooplankton displacement volume) and NMDS scores for two-axis ordination of samples (*: P<0.01, n.s.: not significant).

	Direction cosines			
	X	Y	Adj.R^2^	F
**Temperature (**^**o**^**C)**	0.066	-0.998	32.1	6.45^*^
**Salinity**				0.68^n.s.^
**ZDV (ml m**^**-3**^**)**				0.39^n.s.^

## Discussion

### Larval fish community structure in reef-associated waters

Our study represents the first description of the larval fish communities in the Saudi Arabian waters of the Red Sea, providing information on the temporal differences in abundance and species composition at two distinct sites within an area surrounded by complex coral reef topography. In contrast to the Red Sea, the exploration of the distribution and structure of the early-stage larval fish assemblages in other major tropical coral reef-associated habitats was initiated several decades ago [10–12]. Research efforts have been largely focused in Australian waters and the Pacific [11, 13, 14], the Caribbean [15, 16], the Mesoamerican Barrier Reef [17–20], and in other ecosystems [54, 55]. The majority of these studies have addressed the mesoscale horizontal and/or vertical changes of larval fish communities with changing proximity from coral reefs, while their seasonal variation has been less studied [18, 54–57]. Although the efficiency of molecular taxonomy for larval fish from coral reefs has been tested [32, 33, 58], the ecological characterization of larval fish assemblages so far has been mostly restricted to the family level, with only limited utilization of sequencing techniques [20]. Here, we combined morphological and DNA barcoding approaches to identify larval fish at a sub-family level and to gain further insight on species diversity.

A complex interaction of physical and biological parameters determines the horizontal structure of larval fish communities in reef-associated waters, where larval assemblages of reef species appear in proximity or mixed with oceanic species [11, 20]. Despite the relatively small distance between our sampling stations (~ 8 km), we observed strong spatial differentiation in the larval fish community structure in the cooler period of the year (winter/early spring). This was mainly due to the overwhelming abundance of mesopelagic fish larvae at the deeper offshore site, where the depth requirements of adults (below 200 m during the day) can be satisfied [59]. The observed mesopelagic species assemblage comprised five distinct taxa out of the eight known to form the Red Sea mesopelagic ichthyofauna [21, 60], with *Vinciguerria* sp. and *Benthosema* spp. being the most important representatives. The larvae of *Vinciguerria* sp. (likely the endemic *V*. *mabahiss*) displayed a gradual increase in their abundances from January to April, with much lower abundance during the rest of the year, but it is unclear whether the observed pattern actually reflected the spawning behavior of the adults. Having sampled only the 0–50 m layer, we cannot disregard the possibility that we missed larvae that would have occupied a deeper niche, particularly in the summer stratified water column, avoiding the increased surface temperatures and better satisfying their metabolic demands. Fish larvae show species- and size-specific habitat depth segregations in association with the structure of the water column [61–64], however under stratified conditions larvae of *Benthosema pterotum* and the congeneric species *Vinciguerria nimbaria* seem to be mostly distributed within the mixed layer, at the top of pycnocline or within this stratum [64, 65]. Thus, we believe that our sampling scheme would have likely collected a representative part of the adult spawning output even during the stratification period (start of thermocline at 15–20 m depth).

Contrary to the deeper site, where the dominance of the mesopelagic component was coupled with an infrequent occurrence of reef-associated species, the inshore site during the winter was inhabited by a highly abundant and strikingly diversified gobiid-apogonid species composition. This prominent difference between the sampling stations, however, faded with the seasonal warming of the water column and the larval fish community shifted in both sites towards an enriched larval fish assemblage consisting of numerous species distributed across several reef-associated families. Although the inshore waters supported a high and relatively constant level of diversity all year around, during the colder period the encountered taxa mainly belonged to a few cryptic reef-associated families (e.g., Gobiidae, Apogonidae, Schindleriidae), encompassing small-sized species spawning non-pelagic eggs [10]. A reproductive guild dichotomy within coral reef fishes (i.e., pelagic eggs vs. non-pelagic (demersal, brooded, and viviparous)) seems to largely determine the assemblages of fish larvae near coral reef waters [11]. For instance, larvae of species hatching from non-pelagic eggs are characterized by a more inshore distribution compared to the ones coming from pelagic eggs, although within each reproductive guild this pattern may vary among taxa [66]. Our winter/spring observations seem to support this notion, but during the warmer period larvae of species both with pelagic (e.g., Scaridae, Larbidae, Caesionidae and the majority of the families encountered) and non-pelagic eggs (Gobiidae, Apogonidae, Pomacentridae, Clupeidae), were similarly distributed in the inshore and offshore stations. We assume that this could be mostly related to the short distance to the surrounding reefs, which falls well within the modeled distances for the larval fish dispersal around the study area [26] and well within distances for reef fish larvae [67, 68]. Thus, why larvae of reef fishes were almost absent from the offshore site in February-March (cluster Group A) and what fueled the occurrence of such particular gobiid-apogonid assemblage in the same period in the inshore waters remains an interesting question.

Water temperature appeared to be the main driver of the temporal changes in the structure of the larval fish community in our study, with higher temperatures (after June) signaling the numerical increase of larvae belonging to many families, particularly Scaridae and Labridae, although species-specific variations within a family were also detected (e.g., *Scarus niger* was only present in winter). Seasonality in assemblage structure (e.g., winter vs. summer) of larval fishes has been detected in several studies in other tropical areas where ichthyoplankton was sampled on a monthly [18, 54, 55] or less frequent basis [56, 57] for one or two-year periods. No previous information on larval fish abundance and diversity is available for the Red Sea. However, very recent work exploring the fish recruitment patterns in three distinct coral reefs in proximity to our sampling stations [69, 70], has revealed consistent seasonal recruitment peaks in the fall and early winter for most of the dominant families (i.e., parrotfishes, wrasses, gobiies), in accordance with our observed abundance peaks during the warm period of the year (e.g., Sep, Oct, Nov).

A strong seasonality in phytoplankton biomass has been additionally reported for the central Red Sea [71], involving an abrupt bloom initiation in autumn, peaking in mid-January and terminating in early spring, while an additional peak appears in June, likely associated with the development of short lasting (2–3 weeks) anti-cyclonic eddies in the area [71]. Increased temperatures in the tropics are expected to accelerate bottom-up responses and shorten lag phases among trophic levels. Indeed, our ZDV measurements in the sampling layer followed closely the reported annual phytoplankton patterns, with a notable increase after the summer period. The detected maxima of occurrence of larval fish in several families seemed also to coincide with the rich ZDV period, even showing additional peaks in June, in agreement with the abovementioned short-lasting fueling of the ecosystem [71]. However, the contribution of this environmental parameter to the seasonal changes in the fish community structure as a whole was not significant.

The above mentioned lack of association may be explained in several ways. Although food availability is expected to influence the adult spawning activity and establish initial larval abundances, reef fishes encompass a wide range of feeding modes [72–76] and thus may not show larval production responses to zooplankton peaks. In addition, ZDV measurements were a proxy of zooplankton biomass for organisms larger than 500 μm and excluded smaller-sized prey items that are important for larval fish survival. ZDV changes also do not take into account composition shifts in the zooplankton community and the consequential implications on the food web. For instance, our offshore collections in winter/spring months had a prominent occurrence of gelatinous zooplankton (i.e., medusae, salps) and large-sized chaetognaths. Similarly, the low larval abundance and diversity observed offshore in May coincided with a striking chaetognath dominance and copepod absence in the sample. As both jellyfish and chaetoghaths are known to compete for food resources with larval fish or even prey on them [77–79], the lower species richness at the offshore station during winter/early spring could be associated with complex food web interactions, including predation pressure and competition for prey.

In contrast to the taxa that exhibited seasonality in their distribution, many others showed an extended or year-round presence in both inshore and offshore waters. Sustained reproduction and recruitment has been suggested as a strategy to counteract predation pressure and avoid population extinctions in tiny reef-associated cryptobenthic species with short adult lifespans [80, 81]. This was observed here for several gobiid (e.g., *Eviota*) and apogonid taxa, as well as for the small, pelagic and short-lived clupeoid *Spratelloides gracilis* [less than 4 months; [82]] that is known to reproduce throughout the year in the Indo-Pacific [83, 84]. Surprisingly, however, many other species were encountered only occasionally; this applied to many of the gobiid and apogonid species collected exclusively in early spring from the inshore waters (samples of Group c). Given that such species are expected to reproduce throughout the year it seems rather unlikely that the observed distribution patterns have been generated by temporally restricted spawning preferences of the adults. Recent studies in the area have reported variations among the adult reef communities across the local continental shelf [85–87], as well as a significant differentiation in the current features among reefs, both on a spatial and seasonal basis [88]. These parameters, combined with the species-specific vertical distribution patterns [11, 89] and behavioral traits [90] of larval fish, may result in highly variable dispersal trajectories among taxa that could possibly provide some explanation to our observations.

The clustering of samples at the species level during our study showed a further spatiotemporal variation in the community structure compared to the one observed at the family level. Collections sampled mainly from offshore waters during cooler months were characterized not only by mesopelagic species, but also by a stable non-mesopelagic species assemblage, that would not be evident at the family level. We acknowledge that as this study was limited to a single annual cycle, definitive conclusions about seasonal or inter-annual patterns cannot be drawn. In addition, as our sampling did not include replicates and was not aligned with specific lunar phases this may have introduced some bias in our interpretation and possible underestimation of very rare taxa. However, given the diversity of species captured in our samples and the relatively wide range of developmental stages, we think the results still present a useful picture of the species richness of the larval pool in the area and the spatiotemporal associations among the dominant taxa.

### Filling the biodiversity gap: The benefit of planktonic tows and DNA barcoding

Towed plankton nets, unselectively capturing the early life-history stages of fishes that live in a wide variety of habitats as adults, yield a much higher species diversity compared to other sampling methods that target late-stage larvae [19, 33, 91]. The requirement, however, for expertise in morphological identification of the very early life stages combined with a lack of taxonomic descriptions for all developmental stages [8], has so far discouraged multispecies larval studies using classical taxonomic approaches. Molecular identification of fish larvae, using DNA barcoding, has recently allowed for great advances in the field, and has facilitated the estimation of biodiversity levels and distribution patterns in temperate and tropical marine ecosystems [20, 31].

Recent studies testing the efficacy of DNA barcoding in larvae of tropical latitudes stressed that species identification is limited by the paucity of molecular data available in public databases (especially for certain families) and raised concerns that morphological misidentification of specimens may often lead to erroneous records [30, 33]. Blasting our sequences against the GenBank database resulted in unsuccessful species assignments in many instances, revealing that Red Sea ichthyofauna is largely understudied, particularly for non-commercial families and cryptobenthic species, such as Apogonidae and Gobiidae. Although the number of cataloged fish species in the Red Sea is being continuously amended, in only a few cases has the description of a new species been corroborated using molecular data [92]. An additional comparison of our data with a forthcoming COI-barcode library built based on juvenile and adult fish sampled along Arabian Red Sea reefs only slightly improved the identification to the species level as the majority of collected larvae appeared to be species not commonly seen or sampled as adults. Species diversity encountered in larval samples highly outnumbered the post-metamorphosis records, and even in the cases of exact assignments, many of the species remain to be formally named and described. Although in the worldwide framework to barcode fish species, adult stages could ideally serve as DNA-identifiers for their larvae [40], species identification for many Red Sea taxa still needs to be formalized, even for stages with fully developed morphological characters. Our findings stress the urgent need to construct reliable sequence reference databases for Red Sea species, based on complete and accurate morphological identification of post-metamorphosis stages.

In fourteen cases (S3 and S4 Tables) the species assigned to our barcodes have not been reported to occur in the Red Sea [21], although they are present in the Indo-Pacific [47]. The similarity percentage was high enough and the assignment statistically significant, which suggests that perhaps we should consider the possibility that our data provide information for new records, although these certainly should also be taxonomically verified before the species are formally included in the fish fauna of the Red Sea. Primer suitability and the target gene in DNA barcoding are also critical parameters to be considered in our future efforts for species identification based on molecular data available in public databases. The COI gene and the fish cocktail primers used in this study have been shown to be effective in differentiating among large number of fish taxa [41], but are not always effective for tropical taxa (e.g., the *Schindleria* genus in our study).

Our results underlined the benefits and limitations of COI-barcoding in estimating larval fish diversity and highlighted the importance of a combination of morphological and molecular approaches in future works. Larval fish studies based on net tows in the water column highly complement the estimation of fish biodiversity that is usually based on more spatially constrained methods targeting older stages (e.g., light traps, ichthyocides and visual census). Working towards the development of a database incorporating information from different life stages will provide a comprehensive picture of the species richness of the Red Sea ichthyofauna and will help in future biodiversity conservation and reef ecosystem management plans.

## Supporting information

S1 Fig Assignment at the sub-family level by combined DNA barcoding and morphology. (PDF)

S2 Fig Abundance of encountered families in the samples. (PDF)

S3 Fig Abundance of gobiid species in the samples. (PDF)

S4 Fig Abundance of apogonid species in the samples. (PDF)

S5 Fig Abundance of labrid, pomacentrid and scarid species in the samples. (PDF)

S1 Table Dates of ichthyoplankton sampling. (PDF)

S2 Table Primers used for the DNA barcoding. (PDF)

S3 Table List of non-gobiid taxa identified in the samples. (PDF)

S4 Table List of gobiid taxa identified in the samples. (PDF)
