# Supplementary material for: Exploring the larval fish community of the central Red Sea with an integrated morphological and molecular approach
Source: PLoS One. 2017 Aug 3;12(8):e0182503. doi: 10.1371/journal.pone.0182503 (PMC5542619; doi:10.1371/journal.pone.0182503)
Supplement: S1 Fig — (PDF) [file pone.0182503.s001.pdf]

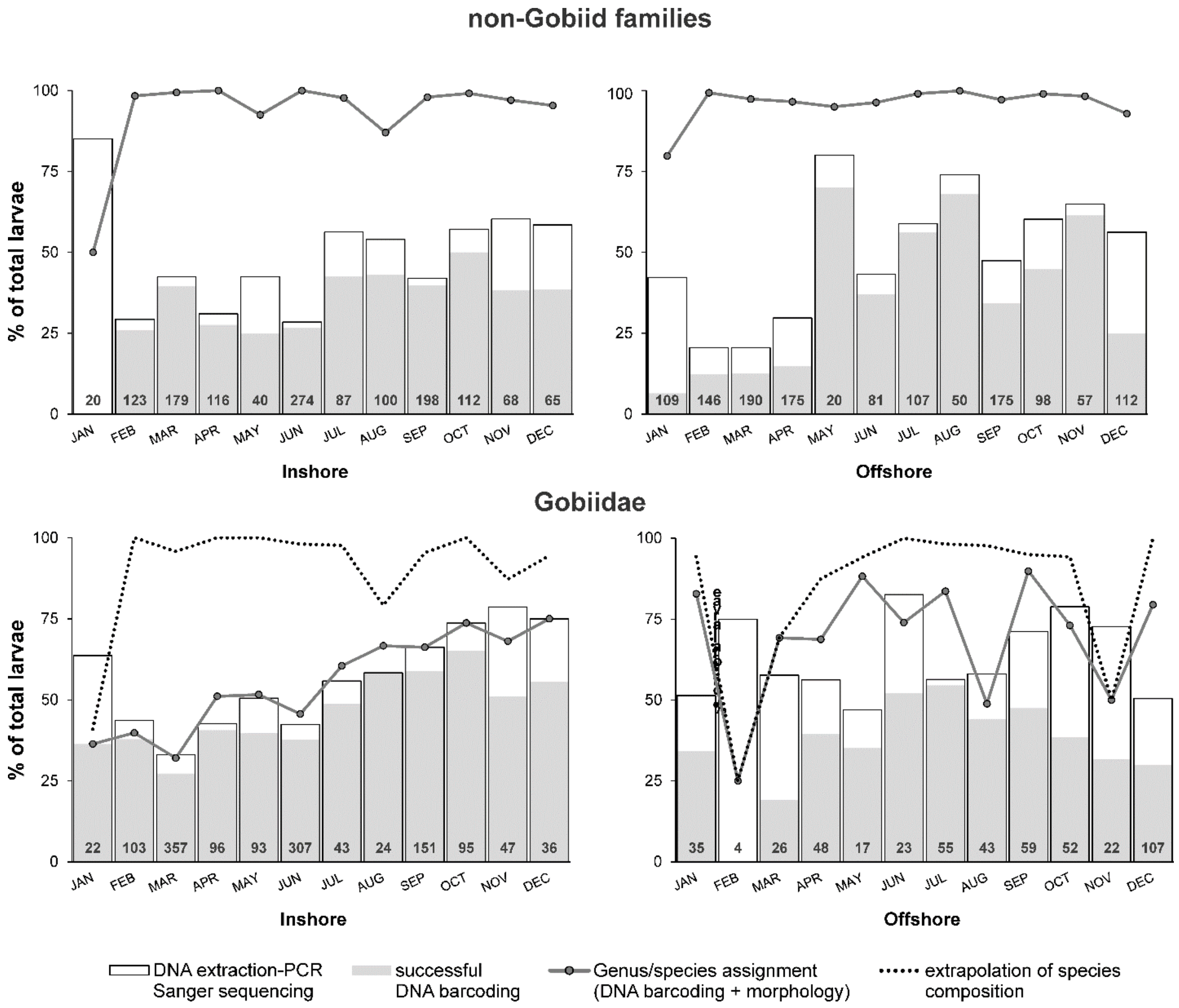

S1 Fig. Assignment at the sub-family level by combined DNA barcoding and morphology. Larvae (%) assigned at the sub-family level (non-gobiid families vs. gobiids) by the combined use of DNA barcoding and morphology (numbers at the column base indicate the larvae sorted out per sample). DNA barcoding was not successful for all the larvae processed molecularly (i.e., DNA extraction-PCR-Sanger Sequencing). Morphological features highly helped the taxonomic identification particularly for non-gobiid larvae, while species composition per gobiid morphotype was extrapolated to the rest of the gobiids of the same morphotype in the sample.
