# Supplementary material for: Exploring the larval fish community of the central Red Sea with an integrated morphological and molecular approach
Source: PLoS One. 2017 Aug 3;12(8):e0182503. doi: 10.1371/journal.pone.0182503 (PMC5542619; doi:10.1371/journal.pone.0182503)
Supplement: S2 Fig — (PDF) [file pone.0182503.s002.pdf]

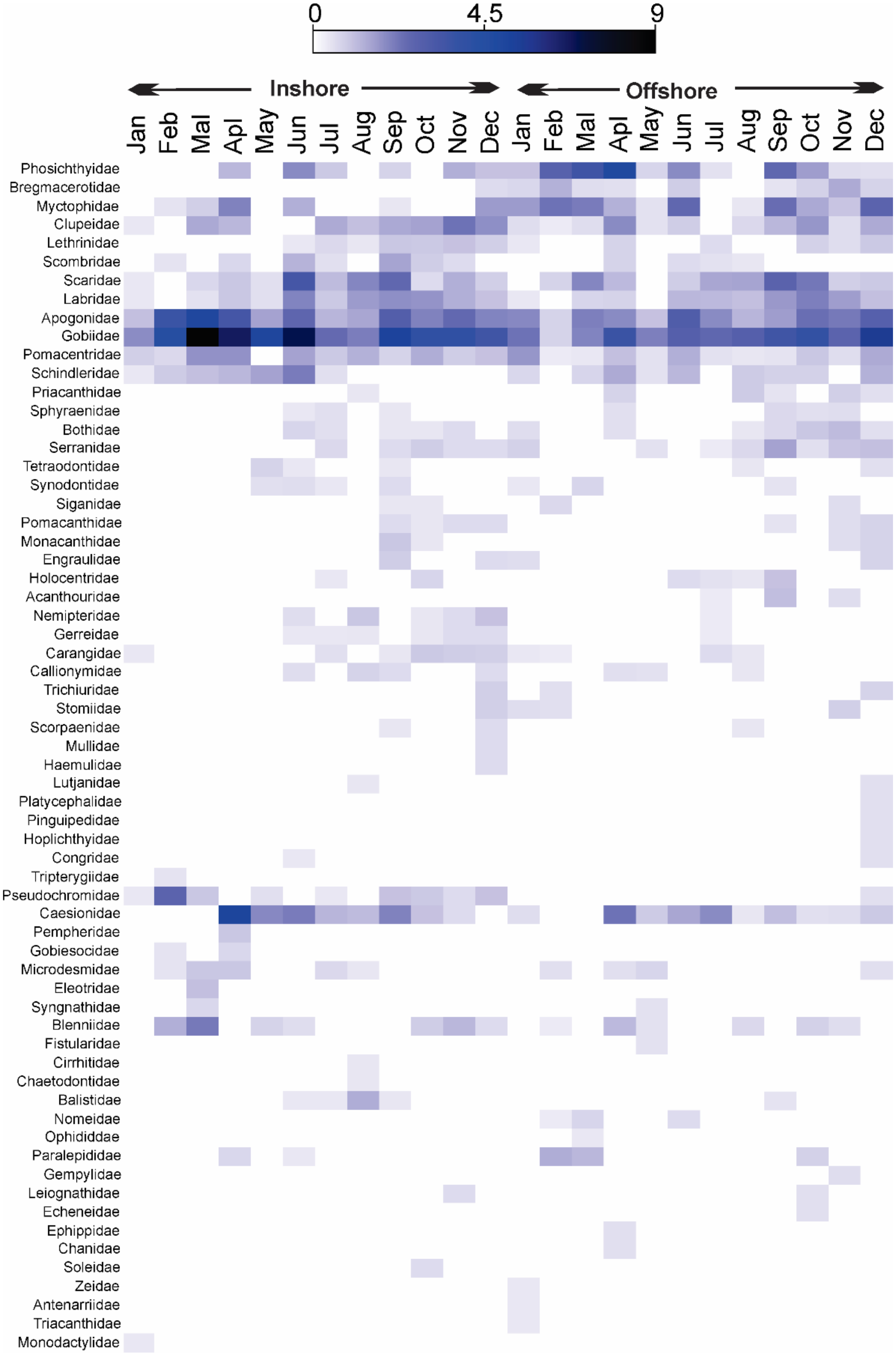

S2 Fig. List of the encountered families. Shade plot of square-root transformed abundances for the families encountered in the monthly collections. Linear color-scale is proportional to the square root transformed abundances of each taxon.
