# Supplementary material for: Exploring the larval fish community of the central Red Sea with an integrated morphological and molecular approach
Source: PLoS One. 2017 Aug 3;12(8):e0182503. doi: 10.1371/journal.pone.0182503 (PMC5542619; doi:10.1371/journal.pone.0182503)
Supplement: S3 Fig — (PDF) [file pone.0182503.s003.pdf]

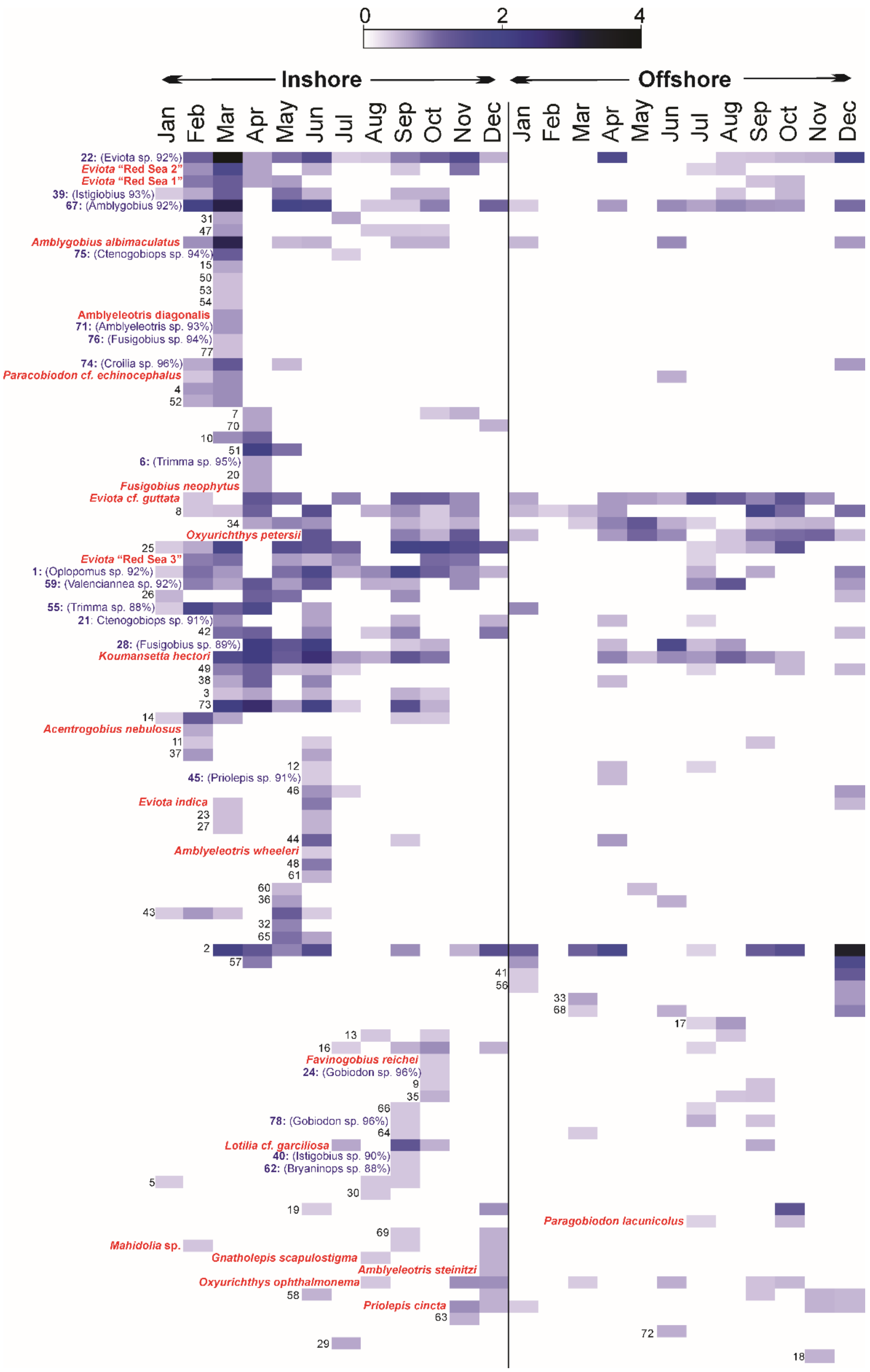

S3 Fig. Abundance of gobiid species in the samples. Shade plot of square-root transformed abundances of the gobiid species encountered in the monthly collections. Linear color-scale is proportional to the square root transformed abundances of each taxon. Taxa identified to species level have been presented in red. Numbers 1-78 correspond to the genetic species Gobiidae 1-78 presented in S4 Table. Taxa that could be identified to genus level according to statistics (see methods) has been presented in blue and the name of the assigned genus is indicated along with the % similarity.
