# Supplementary material for: Exploring the larval fish community of the central Red Sea with an integrated morphological and molecular approach
Source: PLoS One. 2017 Aug 3;12(8):e0182503. doi: 10.1371/journal.pone.0182503 (PMC5542619; doi:10.1371/journal.pone.0182503)
Supplement: S4 Fig — (PDF) [file pone.0182503.s004.pdf]

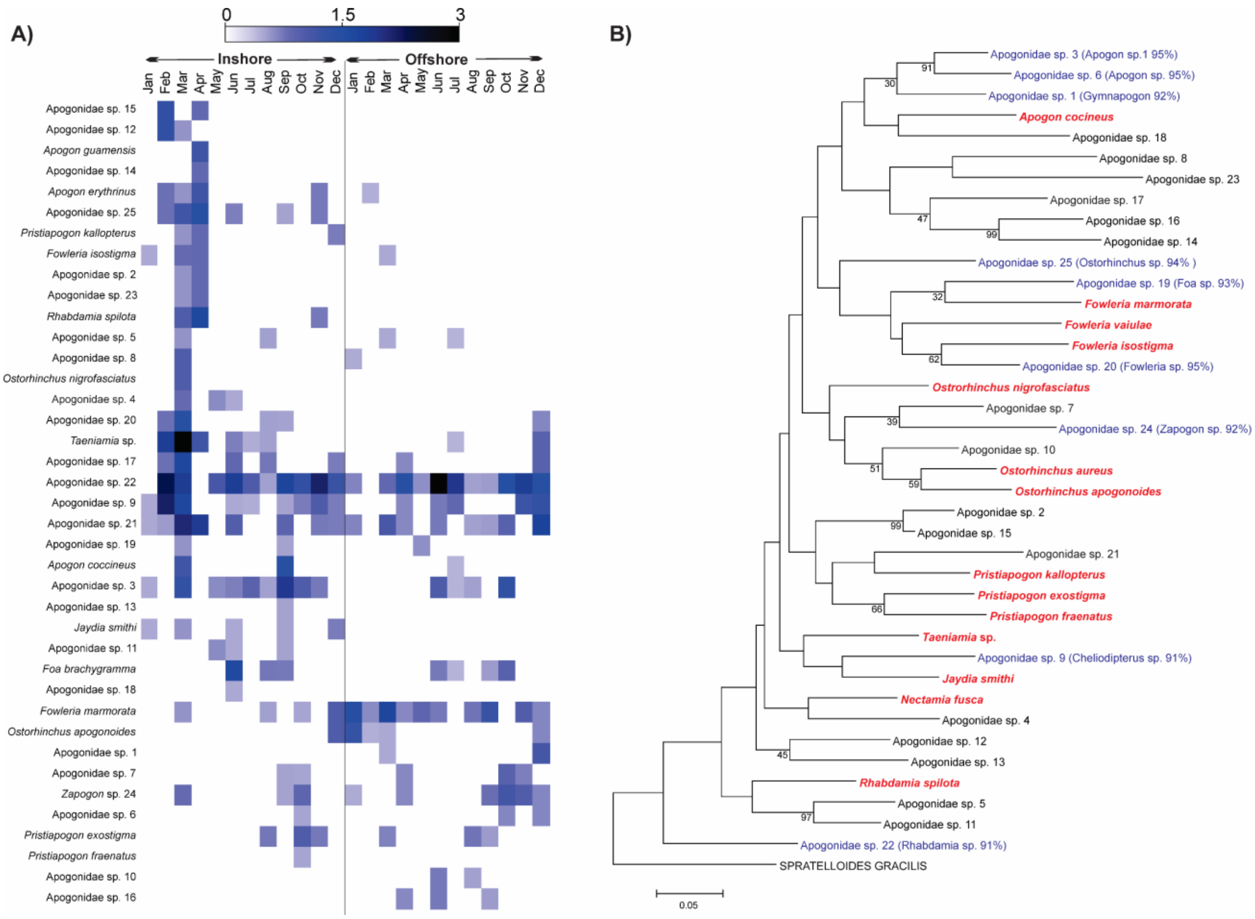

S4 Fig. Abundance of apogonid species in the samples. A) Shade plot of square-root transformed abundances for the species of the family Apogonidae encountered in the monthly collections. Linear color-scale is proportional to the square root transformed abundances of each taxon B) Phylogenetic tree of the family using as outgroup the clupeoid *Spratelloides gracilis*. Taxa identified to species level have been presented in red. Taxa that could be identified to genus level according to statistics (see methods) has been presented in blue and the name of the assigned genus is indicated along with % similarity.
