# Supplementary material for: Exploring the larval fish community of the central Red Sea with an integrated morphological and molecular approach
Source: PLoS One. 2017 Aug 3;12(8):e0182503. doi: 10.1371/journal.pone.0182503 (PMC5542619; doi:10.1371/journal.pone.0182503)
Supplement: S5 Fig — (PDF) [file pone.0182503.s005.pdf]

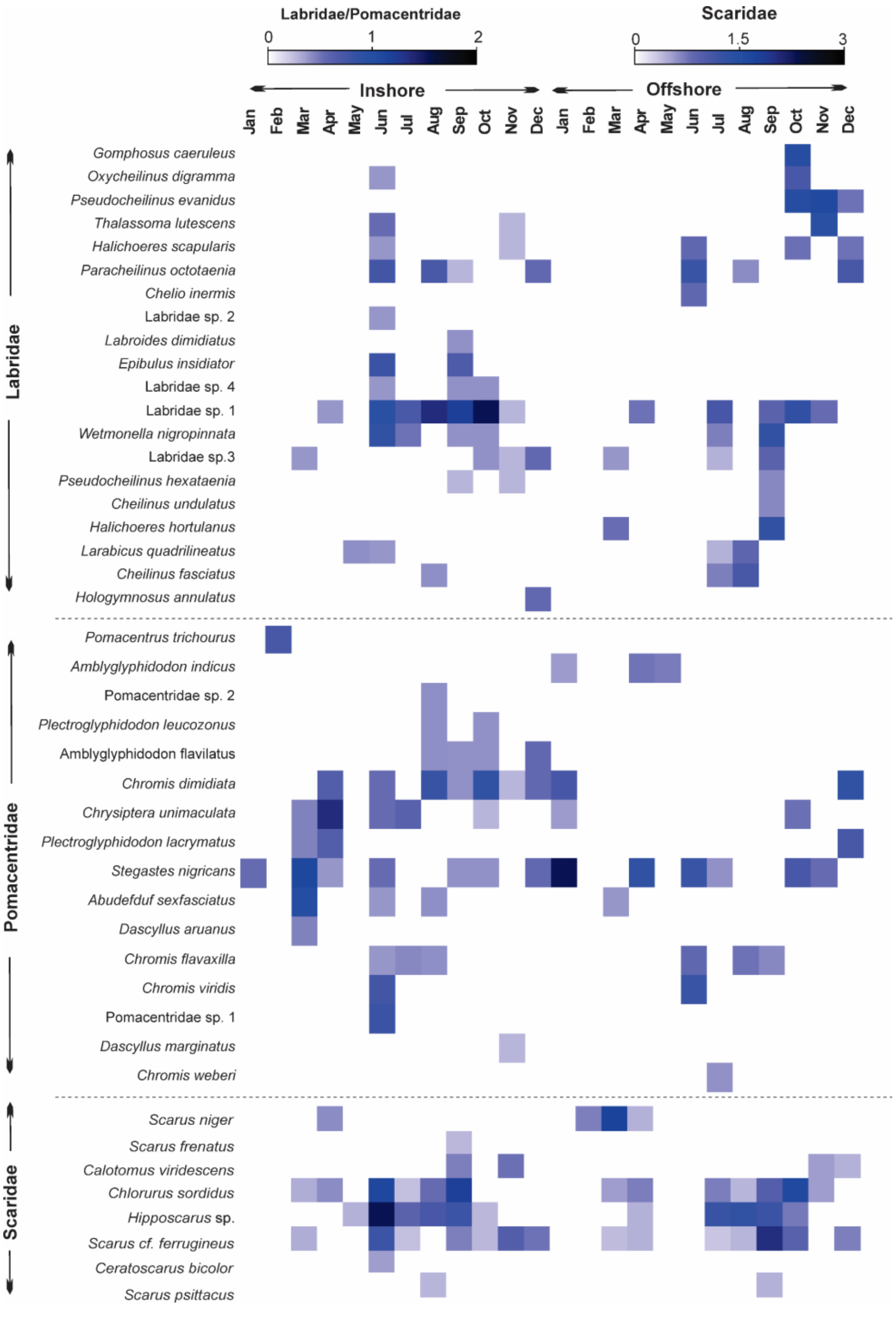

S5 Fig. Abundance of labrid, pomacentrid and scarid taxa in the samples. Shade plot of square-root transformed abundances for the species of the families Labridae, Pomacentridae and Scaridae encountered in the monthly collections. Linear color-scales are proportional to the square root transformed abundances of each taxon (please note the difference in scale for the family Scaridae).
