# Supplementary material for: Exploring the larval fish community of the central Red Sea with an integrated morphological and molecular approach
Source: PLoS One. 2017 Aug 3;12(8):e0182503. doi: 10.1371/journal.pone.0182503 (PMC5542619; doi:10.1371/journal.pone.0182503)
Supplement: S1 Table — (PDF) [file pone.0182503.s006.pdf]

S1 Table. Dates of ichthyoplankton sampling.

	1	2	3	4	5	6	7	8	9	10	11	12	13	14	15	16	17	18	19	20	21	22	23	24	25	26	27	28	29	30	31
Jan					☾						●								☽								○				
Feb			☾							●							☽								○						
Mar					☾						●								☽								○				
Apr			☾							●								☽							○						
May		☾								●								☽							○						☾
Jun								●								☽							○							☾	
Jul								●								☽						○							☾		
Aug							●							☽							○							☾			
Sep					●							☽							○								☾				
Oct					●							☽							○								☾				
Nov					●					☽							○								☾						
Dec			●						☽								○								☾						

Dates of sampling (marked with yellow) compared to the lunar phase (●: new moon, ☽: first quarter, ○: full moon, ☾: third quarter).
