# Supplementary material for: Exploring the larval fish community of the central Red Sea with an integrated morphological and molecular approach
Source: PLoS One. 2017 Aug 3;12(8):e0182503. doi: 10.1371/journal.pone.0182503 (PMC5542619; doi:10.1371/journal.pone.0182503)
Supplement: S2 Table — (PDF) [file pone.0182503.s007.pdf]

S2 Table. Primers used for the DNA barcoding. COI amplification in our study was done by the use of Fish cocktail primers.

**Fish cocktail (M13 tailed): C_FishF1t1–C_FishR1t1 (Ratio 1:1:1:1) [1]**	
VF2_t1	TGTAAAACGACGGCCAGTCAACCAACCACAAAGACATTGGCAC
FishF2_t1	TGTAAAACGACGGCCAGTCGACTAATCATAAAGATATCGGCAC
FishR2_t1	CAGGAAACAGCTATGACACTTCAGGGTGACCGAAGAATCAGAA
FR1d_t1	CAGGAAACAGCTATGACACCTCAGGGTGTCCGAARAAYCARAA

**Sequencing primers for M13-tailed PCR products [2]**	
M13F	TGTAAAACGACGGCCAGT
M13R	CAGGAAACAGCTATGAC

References

1. Ivanova NV, Zemlak TS, Hanner RH, Hebert PDN. Universal primer cocktails for fish DNA barcoding. Mol Ecol Notes. 2007; 7: 544-548. doi: 10.1111/j.1471-8286.2007.01748.x.

2. Messing J. New M13 vectors for cloning. Methods Enzymol. 1983; 101: 20-78.
