# Supplementary material for: Exploring the larval fish community of the central Red Sea with an integrated morphological and molecular approach
Source: PLoS One. 2017 Aug 3;12(8):e0182503. doi: 10.1371/journal.pone.0182503 (PMC5542619; doi:10.1371/journal.pone.0182503)
Supplement: S3 Table — (PDF) [file pone.0182503.s008.pdf]

S3 Table. List of non-gobiid taxa identified in the samples. List of non-gobiid taxa identified in the samples by integrative taxonomy (X: mean annual abundance in larvae m^{-2}, %FO: percentage of frequency of occurrence in each station).Taxa with no occurrence in the Red Sea according to an updated checklist of Red Sea fishes [1] and/or the Red Sea FishBase [2] are indicated with (*). Taxa identified based only on morphological characters are indicated with (M) and comments on species naming are indicated with superscripts.

			Inshore		Offshore	
**Order**	**Family**	**Sub-Family level**	**X**	**FO%**	**X**	**FO%**
Anguiliformes	Congridae	Congridae sp.	0.01	8	-	-
		Uroconger sp.	-	-	0.02	8
Aulopiformes	Paralepididae	*Lestrolepis* sp.	0.05	17	0.29	25
	Synodontidae	*Harpadon squamosus**	0.01	8	0.02	8
		Saurida undosquamis	0.02	8	0.01	8
		Synodus variegatus	0.05	25	0.04	8
Beryciformes	Holocentridae	*Myripristis murdjan*	0.01	8	0.06	17
		Myripristis chryseres	-	-	0.02	8
		Myripristis sp.	0.02	8	0.02	17
		*Ostichthys kaianus**	0.01	8	0.01	8
		Holocentridae sp.	-	-	0.02	8
		Unidentified Holocentridae	-	-	0.02	8
Clupeiformes	Clupeidae	Clupeidae sp.	-	-	0.02	8
		Spratelloides gracilis	1.50	75	0.91	83
	Engraulidae	Engraulidae sp.	0.08	17	0.04	8
		Thryssa baelama	-	-	0.02	8
Gadiformes	Bregmacerotidae	*Bregmaceros* sp.	0.03	8	0.51	75
Gobiesociformes	Gobiesocidae	Gobiesocidae sp. (M)	0.05	17	-	-
Lophiiformes	Antenarriidae	*Antennarius coccineus*	-	-	0.01	8
Myctophiformes	Myctophidae	*Benthosema* spp.	0.73	50	2.78	92
Ophiddiformes	Ophididdae	*Brotula* sp. (M)	-	-	0.01	8
Perciformes	Acanthouridae	*Acanthurus sohal*	-	-	0.08	8
		Ctenochaetus striatus	-	-	0.02	17
		Naso elegans	-	-	0.02	8
	Apogonidae	*Apogon coccineus*	0.17	17	0.01	8
		*Apogon erythrinus**	0.14	33	0.01	8
		Apogon guamensis[1]	0.07	8	-	-
		Apogonidae sp. 1	-	-	0.08	17
		Apogonidae sp. 2	0.05	17	-	-
		Apogonidae sp. 3	0.46	75	0.17	33
		Apogonidae sp. 4	0.07	25	-	-
		Apogonidae sp. 5	0.03	17	0.02	17
		Apogonidae sp. 6	0.01	8	0.04	17
		Apogonidae sp. 7	0.03	17	0.09	25
		Apogonidae sp. 8	0.05	8	0.01	8
		Apogonidae sp. 9	0.53	75	0.24	42
		Apogonidae sp. 10	-	-	0.04	17

S3 Table continued

Order	Family	Taxa	Inshore X	Inshore FO%	Offshore X	Offshore FO%
		Apogonidae sp. 11	0.05	25	-	-
		Apogonidae sp. 12	0.11	17	-	-
		Apogonidae sp. 13	0.01	8	-	-
		Apogonidae sp. 14	0.04	8	-	-
		Apogonidae sp. 15	0.13	17	-	-
		Apogonidae sp. 16	-	-	0.06	25
		Apogonidae sp. 17	0.26	42	0.06	17
		Apogonidae sp. 18	0.01	8	-	-
		Apogonidae sp. 19	0.03	17	0.02	8
		Apogonidae sp. 20	0.16	33	0.02	8
		Apogonidae sp. 21	0.57	67	0.35	67
		Apogonidae sp. 22	1.29	83	1.24	92
		Apogonidae sp. 23	0.05	17	-	-
		Apogonidae sp. 24	0.09	25	0.20	50
		Apogonidae sp. 25	0.11	25	0.07	33
		Fowleria vaiulae	0.19	25	0.10	33
		Fowleria isostigma	0.08	25	0.01	8
		Fowleria marmorata	0.10	33	0.54	83
		Jaydia smithi	0.08	42	-	-
		Ostorhinchus apogonoides	0.05	8	0.12	33
		Ostorhinchus nigrofasciatus	0.05	8	-	-
		Pristiapogon exostigma	0.11	25	0.07	25
		Pristiapogon fraenatus	0.01	8	-	-
		Pristiapogon kallopterus	0.08	25	-	-
		Rhabdamia spilota	0.22	25	-	-
		Taeniamia sp.	0.73	50	0.05	17
		Unidentified apogonids	0.27	50	-	-
	Blenniidae	*Blenniella periophthalmus*	0.07	33	0.11	33
		Blenniidae sp. 1	0.36	25	0.04	8
		Blenniidae sp. 2	0.03	8	-	-
		Blenniidae sp. 3	0.01	8	0.02	8
		Cirripectes castaneus	0.03	8	0.04	17
		Ecsenius nalolo[2]	0.03	8	-	-
		Meiacanthus cf. nigrolineatus	0.10	25	0.01	8
		Petroscirtes mitratus	0.09	17	-	-
		Unidentified blennids	0.03	8	-	-
	Caesionidae	*Caesio lunaris*	0.07	8	0.05	17
		Caesio cf. caerulaurea	3.14	50	1.03	75
		Gymnocaesio gymnoptera	0.03	8	0.05	25
		Pterocaesio sp.	0.01	8	-	-
	Callionymidae	Callionymidae sp. 1	0.01	8	-	-
		Callionynidae sp. 2	-	-	0.02	8

S3 Table continued

Order	Family	Taxa	Inshore		Offshore	
			X	FO%	X	FO%
		Callionymidae sp. 3	0.06	17	0.01	8
		Callionymus filamentosus	0.02	8	-	-
		Unidentified callionymids	0.03	8	0.02	8
	Carangidae	*Atule mate*	-	-	0.01	8
		Carangidae sp. 1	0.03	8	0.02	17
		Carangidae sp. 2 (M)	-	-	0.01	8
		Carangoides fulvoguttatus	0.03	8	-	-
		Alectis ciliaris	0.03	8	-	-
		Carangoides sp.	-	-	0.02	8
		Decapterus russelli	0.03	8	-	-
		Gnathanodon speciosus	0.02	8	-	-
		Megalaspis cordyla	0.01	8	-	-
		Selar crumenophthalmus	0.07	8	-	-
		Unidentified carangids	0.01	8	-	-
	Chaetodontidae	Chaetodontidae sp.	0.01	8	-	-
	Chanidae	Chanidae sp. (M)	-	-	0.02	8
	Cirrhitidae	*Cirrhitus spilotoceps*[3]	0.01	8	-	-
	Echeneidae	*Echeneidae* sp.	-	-	0.02	8
	Ephippidae	*Platax orbicularis*	-	-	0.02	8
	Gempylidae	*Neoepinnula orientalis**	-	-	0.02	8
	Gerreidae	*Gerres oyena*	0.10	50	0.01	8
	Haemulidae	*Pomadasys stridens*	0.03	8	-	-
	Labridae	*Cheilinus fasciatus*	0.01	8	0.06	17
		Cheilinus undulatus	-	-	0.02	8
		Chelio inermis	-	-	0.03	8
		Epibulus insidiator	0.08	17	-	-
		Gomphosus caeruleus	-	-	0.09	8
		Halichoeres hortulanus	-	-	0.09	17
		Halichoeres scapularis	0.04	17	0.07	25
		Hologymnosus annulatus	0.03	8	-	-
		Labridae sp. 1	0.68	58	0.19	42
		Labridae sp. 2	0.01	8	-	-
		Labridae sp. 3	0.08	33	0.05	25
		Labridae sp. 4	0.04	25	-	-
		Labroides dimidiatus	0.01	8	-	-
		Larabicus quadrilineatus	0.03	17	0.04	17
		Oxycheilinus digramma	0.01	8	0.04	8
		Paracheilininus octotaenia	0.14	33	0.11	25
		Pseudocheiinus hexataenia	0.05	17	0.02	8
		Pseudocheilinus evanidus	-	-	0.21	25
		*Thalassoma lutescens**	0.05	17	0.07	8
		Wetmorella nigropinnata	0.11	33	0.08	17

S3 Table continued

Order	Family	Taxa	Inshore		Offshore	
			X	FO%	X	FO%
		Unidentified labrids	0.01	8	0.05	25
	Leiognathidae	Leiognathidae sp.	-	-	0.02	8
		Photopectoralis bindus	0.03	8	-	-
	Lethrinidae	*Lethrinus harak*	0.02	17	0.04	8
		Lethrinus lentjan	0.03	17	0.05	17
		Lethrinus mahsena	0.03	8	0.04	8
		Lethrinus obsoletus	0.04	8	0.01	8
		Lethrinus sp. 1	0.08	17	0.03	17
		Lethrinus sp. 2	-	-	0.01	8
		Lethrinus spp.	0.12	25	0.03	17
	Lutjanidae	*Lutjanus ehrenbergii*	0.01	8	-	-
		Lutjanus fulviflamma	-	-	0.02	8
	Microdesmidae	*Gunnellichthys monostigma*	0.02	8	-	-
		*Navigobius vittatus**	0.03	17	0.04	8
		Ptereleotris evides	0.03	8	-	-
		*Ptereleotris monoptera**	0.15	42	0.08	25
		Microdesmidae sp.	-	-	0.02	8
	Monodactylidae	Monodactylidae sp. (M)	0.01	8	-	-
	Mullidae	*Upeneus luzonius**[4]	0.03	8	-	-
	Nemipteridae	*Nemipterus bipunctatus*	0.03	8	-	-
		Nemipterus japonicus	0.10	25	-	-
		Scolopsis ghanam	0.01	8	0.01	8
		Nemipteridae spp.	0.07	8	-	-
	Nomeidae	*Cubiceps* sp.*	-	-	0.06	17
	Pempheridae	*Parapriacanthus ransonneti*	0.07	8	-	-
	Pinguipedidae	*Parapercis hexophtalma*	-	-	0.02	8
	Pomacanthidae	*Centropyge multispinis*	-	-	0.02	8
		Genicanthus caudovittatus	-	-	0.02	8
		Pomacanthidae *sp.*	0.09	33	-	-
		Pygoplites diacanthus	-	-	0.05	17
	Pomacentridae	*Abudefduf sexfasciatus*	0.11	25	0.01	8
		Amblyglyphidodon indicus	-	-	0.05	25
		Amblyglyphidodon flavilatus	0.07	33	-	-
		Chromis dimidiata	0.25	58	0.11	17
		Chromis flavaxilla	0.04	25	0.06	25
		Chromis viridis	0.05	8	0.05	8
		Chromis weberi	-	-	0.01	8
		Chrysiptera unimaculata	0.20	42	0.03	17
		Dascyllus aruanus	0.02	8	-	-
		Dascyllus marginatus	0.03	8	-	-
		Plectroglyphidodon lacrymatus	0.05	17	0.04	8
		Plectroglyphidodon leucozonus	0.03	17	-	-

S3 Table continued

Order	Family	Taxa	Inshore X	Inshore FO%	Offshore X	Offshore FO%
		Pomacentrus trichourus	0.03	8	-	-
		Stegastes nigricans	0.27	58	0.31	50
		Pomacentridae sp. 1	0.04	8	-	-
		Pomacentridae sp. 2	0.06	25	0.10	33
		Unidentified pomacentrids	0.01	8	-	-
	Priacanthidae	*Priacanthus hamrur*	-	-	0.04	8
		Priacanthidae sp.	0.01	8	0.11	17
		Unidentified priacanthids	-	-	0.04	17
	Pseudochromidae	*Pseudochromis olivaceus*	0.33	17	-	-
		Chlidichthys auratus	0.21	42	0.02	8
		Pseudochromidae sp. 1	0.03	8	-	-
		Pseudochromidae sp. 2	0.20	25	-	-
		Pseudochromidae sp. 3	0.15	17	-	-
	Scaridae	*Calotomus viridescens*	0.09	17	0.05	17
		Cetoscarus bicolor	0.02	8	-	-
		Chlorurus sordidus	0.64	50	0.43	58
		Hipposcarus sp.	0.79	50	0.46	42
		Scarus frenatus	0.01	8	-	-
		Scarus niger	0.04	8	0.31	25
		Scarus psittacus	0.01	8	0.02	8
		Scarus cf. ferrugineus	0.33	58	0.55	58
		Scarus spp.	0.06	8	-	-
		Unidentified scarids	0.07	25	0.08	25
	Schindleriidae	*Schindleria* sp. (M)	0.82	58	0.62	75
	Scombridae	*Grammatorcynus bilineatus*	0.05	17	-	-
		Katsuwonus pelamis	0.01	8	0.02	8
		Rastrelliger kanagurta	0.33	33	0.06	25
		Scomberomorus commerson	0.03	8	0.02	8
		Scombridae sp. (M)	0.02	8	-	-
		Thunnus tonggol	0.01	8	-	-
	Serranidae	*Cephalopholis hemistiktos*	0.07	25	0.12	25
		Liopropoma lunulatum	-	-	0.02	8
		Liopropoma sp.	-	-	0.03	17
		Pseudanthias squamipinnis	0.03	8	0.19	33
		Serranidae sp. 1	0.04	8	0.06	8
		Serranidae sp. 2	-	-	0.02	8
		Serranidae sp. 3	0.03	8	0.02	8
		Unidentified serranids	-	-	0.01	8
	Siganidae	*Siganus luridus*	0.03	17	0.03	8
		Siganidae sp. (M)	-	-	0.02	8
	Sphyraenidae	*Sphyraena jello*	0.02	8	-	-
		Sphyraena qenie	0.03	17	0.08	25

S3 Table continued

Order	Family	Taxa	Inshore X	Inshore FO%	Offshore X	Offshore FO%
		Sphyraena spp.	-	-	0.02	8
	Trichiuridae	*Tentoriceps* sp.	-	-	0.02	8
		Trichiurus sp.	0.05	8	0.04	17
	unind. Perciformes		0.07	42	0.21	25
Pleuronectiformes	Bothidae	*Arnoglossus* sp.	0.01	8	0.01	8
		Bothidae sp. 1	0.03	17	0.09	17
		Bothidae sp. 2	0.01	8	0.10	17
		Bothidae sp. 3	0.04	17	-	-
		Bothidae sp. 4	-	-	0.02	8
		Bothidae sp. 5	0.02	8	-	-
		Unidentified bothids	-	-	0.04	17
	Soleidae	*Pardachirus marmoratus*	0.01	8	-	-
		Soleidae sp.	0.01	8	-	-
Scorpaeniformes	Hoplichthyidae	*Hoplichthys* sp.*(M)	-	-	0.02	8
	Platycephalidae	Platycephalidae sp. (M)	-	-	0.02	8
	Scorpaenidae	*Scorpaenopsis longispina*	-	-	0.01	8
		Scorpaenidae sp.	0.03	8	-	-
		Unidentified scorpaenids	0.01	8	-	-
Stomiiformes	Phosichthyidae	*Vinciguerria* sp.	0.69	50	4.45	92
	Stomiidae	*Astronesthes* sp.	0.05	8	0.08	25
		Stomiidae sp. (M)	-	-	0.01	8
Syngnathiformes	Fistularidae	*Fistularia commensonnii*	-	-	0.02	8
	Syngnathidae	Syngnathidae sp. 1	0.03	8	-	-
		Syngnathidae sp. 2	-	-	0.02	8
Tetraodontiformes	Balistidae	*Balistoides viridescens*	0.01	8	-	-
		Odonus niger	0.03	17	-	-
		*Sufflamen chrysopterum**	0.14	17	0.02	8
	Monacanthidae	*Cantherhines pardalis*	-	-	0.02	8
		Pervagor cf. randalli	0.08	17	-	-
		Monacanthidae sp. (M)	-	-	0.02	8
		Unidentified monacanthids	-	-	0.02	8
	Tetraodontidae	*Arothron diadematus*	-	-	0.01	8
		*Canthigaster solandri**	0.05	25	0.02	8
		Tetraodontidae sp.	0.02	8	-	-
	Triacanthidae	Triacanthidae sp. (M)	-	-	0.01	8
Zeiformes	Zeidae	Zeidae sp. (M)	-	-	0.01	8
Unidentified fish larvae			0.11	25	0.05	17

[1]corresponds to *Nectamia fusca* according to WoRMs
[2] likely corresponds to *Ecsenius dentex* according to Golani and Bogorodsky (1)
[3] accepted as *Cirrhitus pinnulatus* according to WoRMs
[4] accepted as *Upeneus sundaicus* according to WoRMs
[5] likely the endemic species *Vinciguerria mabahis*

References

1. Golani D, Bogorodsky SV. The fishes of the Red Sea - Reappraisal and updated checklist.

Zootaxa. 2010: 1-100. PubMed PMID: WOS:000277812600001.

2. Froese R, Pauly D. FishBase. World Wide Web electronic publication. www.fishbase.org, version (06/2016). 2016.
