# Supplementary material for: Exploring the larval fish community of the central Red Sea with an integrated morphological and molecular approach
Source: PLoS One. 2017 Aug 3;12(8):e0182503. doi: 10.1371/journal.pone.0182503 (PMC5542619; doi:10.1371/journal.pone.0182503)
Supplement: S4 Table — (PDF) [file pone.0182503.s009.pdf]

**S4 Table. List of gobiid taxa identified in the samples.** Gobiid taxa identified by integrative taxonomy (X: mean annual abundance in larvae $m^{-2}$, %FO frequency of occurrence in each station).Taxa with no occurrence in the Red Sea according to an updated checklist of Red Sea fishes [1] and/or the Red Sea FishBase [2] are indicated with (*). Comments on species naming are indicated with superscripts.

| | Inshore | | Offshore | |
|---|---|---|---|---|
| **Taxon** | **X** | **%FO** | **X** | **%FO** |
| *Acentrogobius nebulosus* | 0.03 | 8 | - | - |
| *Amblyeleotris diagonalis* | 0.04 | 8 | - | - |
| *Amblyeleotris steinitzi* | 0.03 | 8 | - | - |
| *Amblyeleotris wheeleri* | 0.01 | 8 | - | - |
| *Amblygobius albimaculatus* | 0.87 | 50 | 0.12 | 25 |
| *Eviota cf. guttata* | 0.56 | 58 | 0.69 | 75 |
| *Eviota indica* | 0.09 | 17 | 0.02 | 8 |
| *Eviota* sp. 'Red Sea 1' | 0.27 | 33 | 0.04 | 17 |
| *Eviota* sp. 'Red Sea 2' | 0.43 | 50 | 0.02 | 17 |
| *Eviota* sp. 'Red Sea 3' | 0.44 | 58 | 0.01 | 8 |
| *Favonigobius reichei* | 0.01 | 8 | - | - |
| *Fusigobius neophytus* | 0.04 | 8 | - | - |
| *Gnatholepis scapulostigma*[1] | 0.04 | 17 | - | - |
| *Koumansetta hectori* | 1.53 | 67 | 0.35 | 58 |
| *Lotilia cf. garaciliosa* | 0.20 | 25 | 0.03 | 8 |
| *Mahidolia* sp. | 0.05 | 25 | - | - |
| *Oxyurichthys ophthalmonema** | 0.12 | 25 | 0.08 | 33 |
| *Oxyurichthys petersii* | 0.37 | 33 | 0.51 | 75 |
| *Paragobiodon cf .echinocephalus* | 0.07 | 17 | 0.03 | 8 |
| *Paragobiodon lacunicolus** | - | - | 0.03 | 17 |
| *Priolepis cincta* | 0.08 | 17 | 0.06 | 25 |
| Gobiidae sp. 1 | 0.99 | 92 | 0.12 | 25 |
| Gobiidae sp. 2 | 1.00 | 58 | 1.71 | 58 |
| Gobiidae sp. 3 | 0.12 | 42 | - | - |
| Gobiidae sp. 4 | 0.10 | 17 | - | - |
| Gobiidae sp. 5 | 0.04 | 25 | - | - |
| Gobiidae sp. 6 | 0.04 | 8 | - | - |
| Gobiidae sp. 7 | 0.07 | 25 | - | - |
| Gobiidae sp. 8 | 0.46 | 67 | 0.55 | 58 |
| Gobiidae sp. 9 | 0.01 | 8 | 0.02 | 8 |
| Gobiidae sp. 10 | 0.16 | 17 | - | - |
| Gobiidae sp. 11 | 0.03 | 17 | 0.02 | 8 |
| Gobiidae sp. 12 | 0.01 | 8 | 0.03 | 17 |
| Gobiidae sp. 13 | 0.03 | 17 | 0.01 | 8 |
| Gobiidae sp. 14 | 0.20 | 42 | - | - |
| Gobiidae sp. 15 | 0.03 | 8 | - | - |

S4 Table continued

| Taxon | Inshore | | Offshore | |
|---|---|---|---|---|
| | X | %FO | X | %FO |
| Gobiidae sp. 16 | 0.11 | 33 | 0.01 | 8 |
| Gobiidae sp. 17 | - | - | 0.05 | 17 |
| Gobiidae sp. 18 | - | - | 0.02 | 8 |
| Gobiidae sp. 19 | 0.06 | 17 | 0.15 | 8 |
| Gobiidae sp. 20 | 0.04 | 8 | - | - |
| Gobiidae sp. 21 | 0.14 | 33 | 0.03 | 17 |
| Gobiidae sp. 22 | 2.14 | 92 | 0.59 | 50 |
| Gobiidae sp. 23 | 0.04 | 17 | - | - |
| Gobiidae sp. 24 | 0.01 | 8 | - | - |
| Gobiidae sp. 25 | 1.70 | 83 | 0.21 | 33 |
| Gobiidae sp. 26 | 0.26 | 33 | 0.02 | 8 |
| Gobiidae sp. 27 | 0.04 | 17 | - | - |
| Gobiidae sp. 28 | 0.94 | 42 | 0.30 | 33 |
| Gobiidae sp. 29 | 0.03 | 8 | - | - |
| Gobiidae sp. 30 | 0.01 | 8 | - | - |
| Gobiidae sp. 31 | 0.07 | 17 | - | - |
| Gobiidae sp. 32 | 0.06 | 8 | - | - |
| Gobiidae sp. 33 | - | - | 0.08 | 17 |
| Gobiidae sp. 34 | 0.21 | 50 | 0.31 | 67 |
| Gobiidae sp. 35 | 0.03 | 8 | 0.03 | 17 |
| Gobiidae sp. 36 | 0.04 | 8 | 0.03 | 8 |
| Gobiidae sp. 37 | 0.08 | 17 | - | - |
| Gobiidae sp. 38 | 0.20 | 25 | 0.02 | 8 |
| Gobiidae sp. 39 | 0.32 | 58 | 0.04 | 17 |
| Gobiidae sp. 40 | 0.01 | 8 | - | - |
| Gobiidae sp. 41 | - | - | 0.14 | 17 |
| Gobiidae sp. 42 | 0.41 | 50 | 0.02 | 8 |
| Gobiidae sp. 43 | 0.21 | 42 | - | - |
| Gobiidae sp. 44 | 0.12 | 17 | 0.04 | 8 |
| Gobiidae sp. 45 | 0.01 | 8 | 0.02 | 8 |
| Gobiidae sp. 46 | 0.06 | 17 | 0.04 | 8 |
| Gobiidae sp. 47 | 0.08 | 33 | - | - |
| Gobiidae sp. 48 | 0.07 | 8 | - | - |
| Gobiidae sp. 49 | 0.23 | 42 | 0.05 | 25 |
| Gobiidae sp. 50 | 0.02 | 8 | - | - |
| Gobiidae sp. 51 | 0.43 | 17 | - | - |
| Gobiidae sp. 52 | 0.08 | 17 | - | - |
| Gobiidae sp. 53 | 0.02 | 8 | - | - |
| Gobiidae sp. 54 | 0.02 | 8 | - | - |
| Gobiidae sp. 55 | 0.60 | 42 | 0.06 | 8 |
| Gobiidae sp. 56 | - | - | 0.05 | 17 |
| Gobiidae sp. 57 | 0.07 | 8 | 0.26 | 17 |

| S4 Table continued | Inshore | | Offshore | |
|---|---|---|---|---|
| **Taxon** | **X** | **%FO** | **X** | **%FO** |
| Gobiidae sp. 58 | 0.05 | 17 | 0.06 | 25 |
| Gobiidae sp. 59 | 0.51 | 67 | 0.24 | 25 |
| Gobiidae sp. 60 | 0.02 | 8 | 0.02 | 8 |
| Gobiidae sp. 61 | 0.02 | 8 | - | - |
| Gobiidae sp. 62 | 0.01 | 8 | - | - |
| Gobiidae sp. 63 | 0.03 | 8 | - | - |
| Gobiidae sp. 64 | 0.01 | 8 | 0.01 | 8 |
| Gobiidae sp. 65 | 0.12 | 17 | - | - |
| Gobiidae sp. 66 | 0.01 | 8 | 0.01 | 8 |
| Gobiidae sp. 67 | 1.83 | 67 | 0.38 | 67 |
| Gobiidae sp. 68 | - | - | 0.10 | 25 |
| Gobiidae sp. 69 | 0.04 | 17 | - | - |
| Gobiidae sp. 70 | 0.06 | 17 | - | - |
| Gobiidae sp. 71 | 0.04 | 8 | - | - |
| Gobiidae sp. 72 | - | - | 0.03 | 8 |
| Gobiidae sp. 73 | 1.53 | 58 | - | - |
| Gobiidae sp. 74 | 0.19 | 25 | 0.04 | 8 |
| Gobiidae sp. 75 | 0.13 | 17 | - | - |
| Gobiidae sp. 76 | 0.02 | 8 | - | - |
| Gobiidae sp. 77 | 0.02 | 8 | - | - |
| Gobiidae sp. 78 | 0.01 | 8 | 0.04 | 17 |

[1] accepted as *Gnatholepis cauerensis* according to WoRMs

## References

1. Golani D, Bogorodsky SV. The fishes of the Red Sea - Reappraisal and updated checklist. Zootaxa. 2010;(2463):1-100. PubMed PMID: WOS:000277812600001.

2. Froese R, Pauly D. FishBase. World Wide Web electronic publication. www.fishbase.org, version (06/2016). 2016.
